# Supplementary figures and images for: Cell-autonomous requirement for ACE2 across organs in lethal mouse SARS-CoV-2 infection
Source: PLoS Biol. 2023 Feb 6;21(2):e3001989. doi: 10.1371/journal.pbio.3001989 (PMC9934376; doi:10.1371/journal.pbio.3001989)

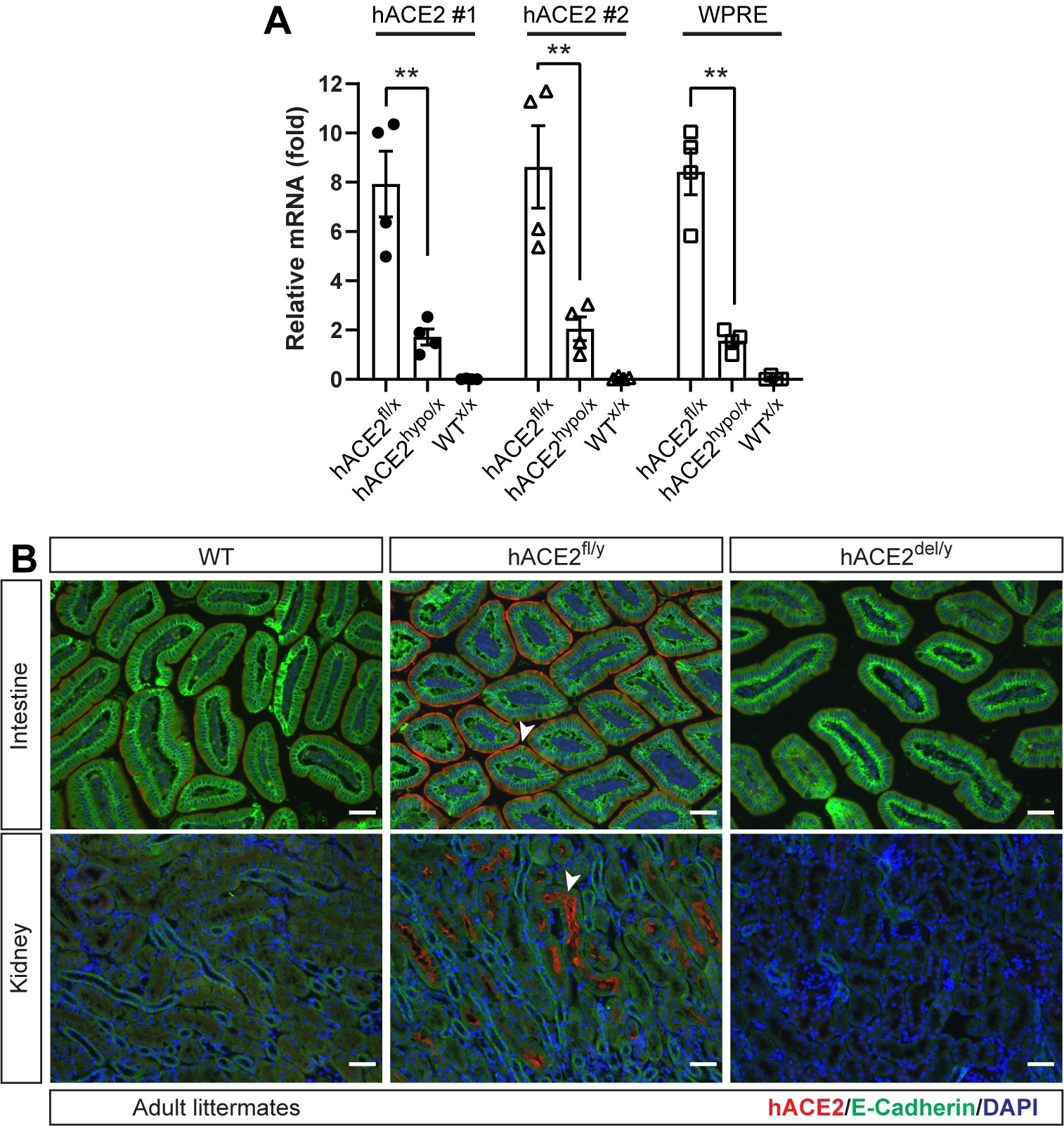

Supplement: S1 Fig — (A) qPCR was performed on total RNA from whole lung from the indicated mice using primers specific for either the hACE2 cDNA (hACE2 #1 and hACE2 #2) or the WPRE cassette **, p < 0.01 by unpaired, two-tailed t test. (B) Immunohistochemistry to detect ACE2 using human-specific ACE2 (hACE2) in the intestine and kidney of wild-type, hACE2fl/y, and hACE2del/y mice. Costaining for the epithelial cell marker E-cadherin. Arrowheads indicate ACE2 staining at the brush border of the intestine (top) and in renal tubular epithelium (bottom). Representative of N = 3 per genotype. Scale bars 50 μm. Numerical data in corresponding Figure Data tab. ACE2, angiotensin-converting enzyme 2; hACE2, human ACE2; WPRE, woodchuck hepatitis virus posttranscriptional regulatory element. (TIF) [file pbio.3001989.s001.tif]

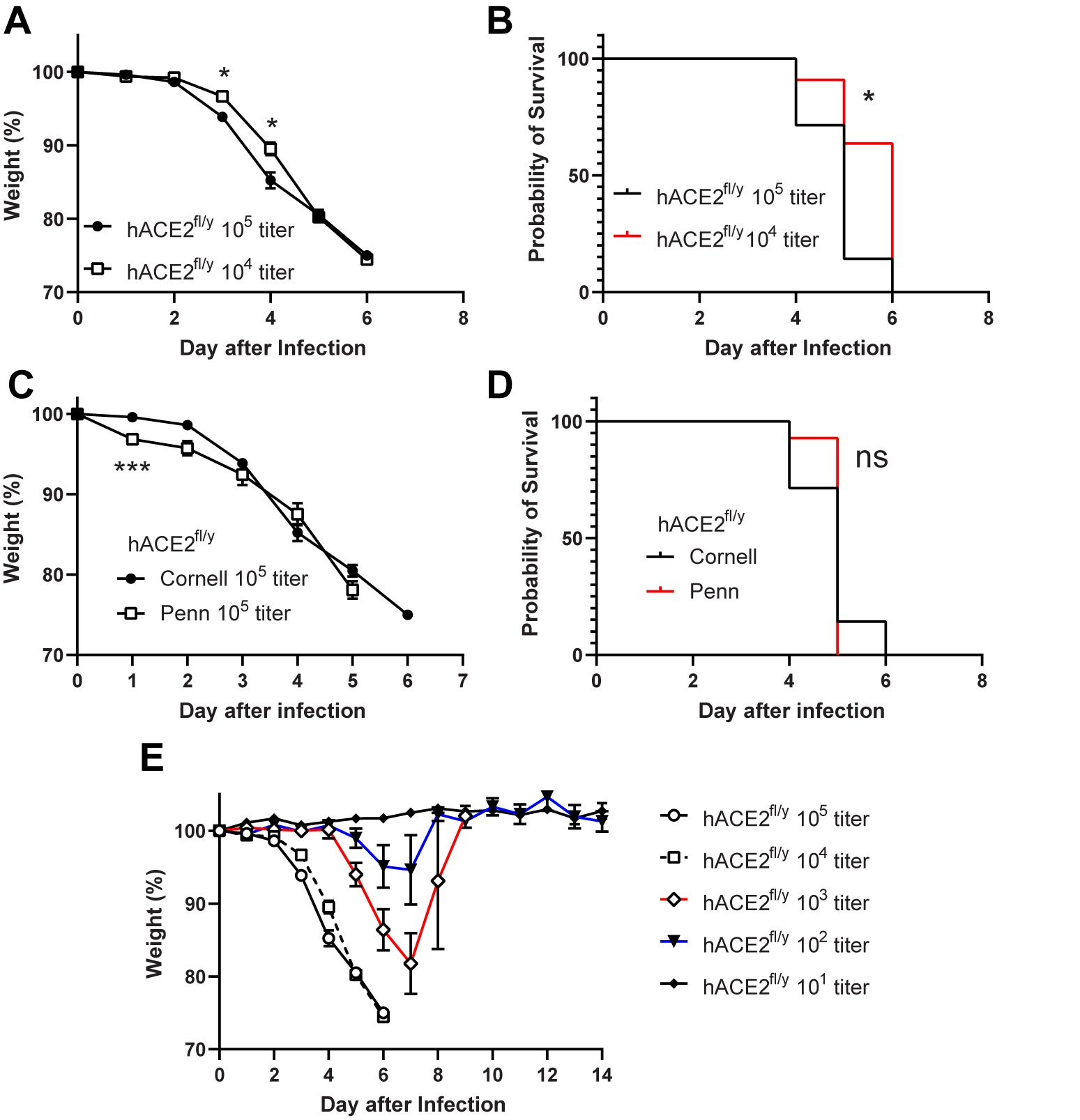

Supplement: S2 Fig — (A-C) Weight loss and survival of hACE2fl/y mice were measured after infection with the indicated PFU of SARS-CoV-2 virus. N = 8 and 11 for 105 and 104 shown in A and B. N = 8, 11, 8, 8, and 6 for 105, 104, 103, 102, and 101 PFU in C. (D, E) Weight loss and survival of hACE2fl/y mice were measured after infection with the indicated PFU of SARS-CoV-2 virus at the Cornell or Penn ABSL3 facilities. N = 8 (Cornell) and 14 (Penn) from 3 independent experiments. Note: The data shown in panels A, B, and E are the same hACE2fl/y data shown in Figs 2 and 4. The Cornell data shown in panels C and D are the same hACE2fl/y data shown in Figs 2 and S6. *p < 0.05; ***p < 0.001; determined by unpaired, two-tailed t-test or log-rank Mantel Cox test. Numerical data in corresponding S1 Metadata tab. ABSL3, Animal Biosafety Level 3; PFU, plaque-forming unit; SARS-CoV-2, Severe Acute Respiratory Syndrome Coronavirus 2. (TIF) [file pbio.3001989.s002.tif]

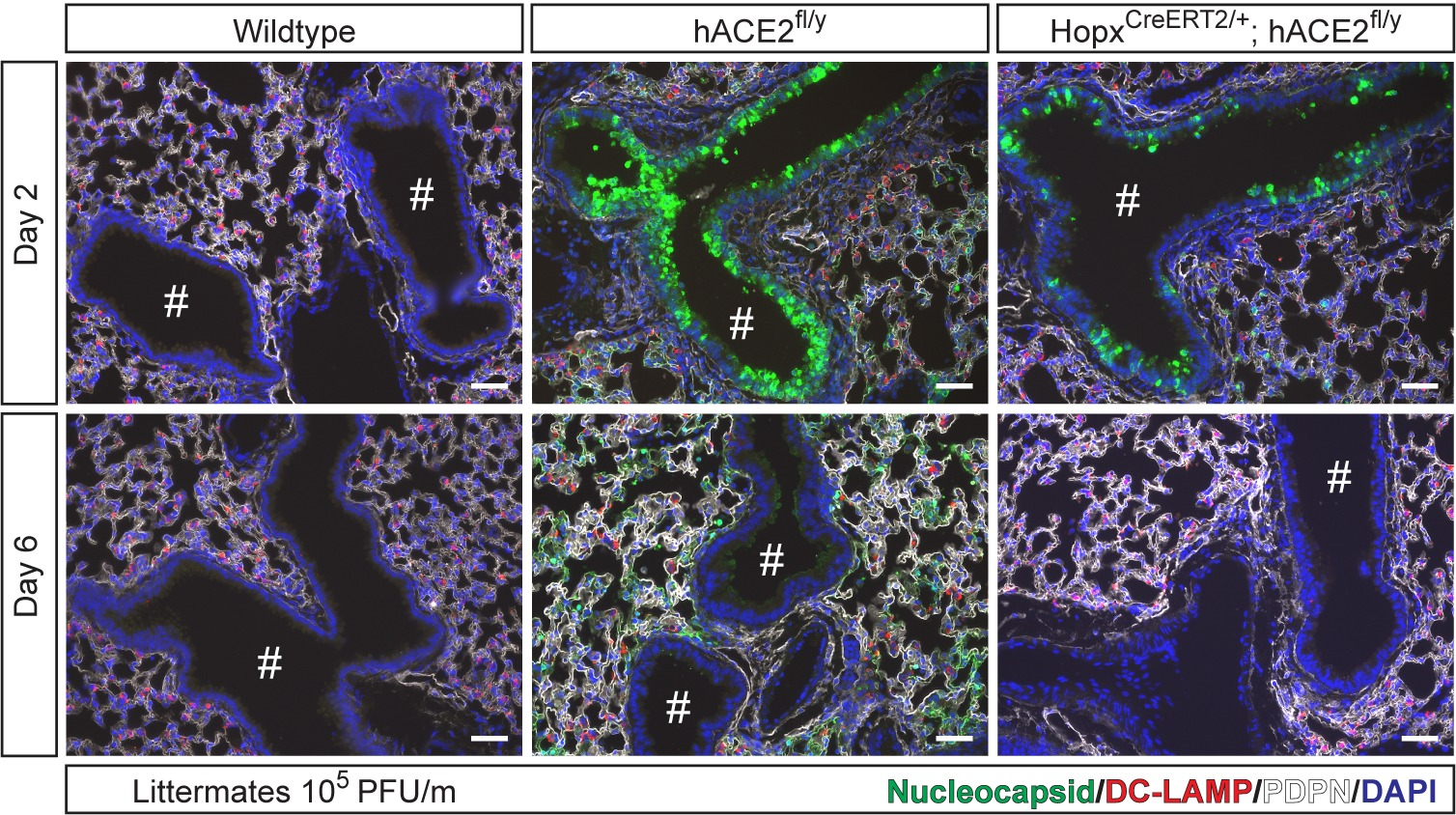

Supplement: S3 Fig — Immunohistochemistry of SARS-CoV-2 nucleocapsid, AT1 cell PDPN, and AT2 cell DC-LAMP in the lung 2 (top) and 6 (bottom) days after infection of wild-type, hACE2fl/y, and HopxCreERT2; hACE2fl/y mice. Hashtags in the center of bronchi. Representative of N = 4–5 animals per genotype and time point. Scale bars 50 μm. AT1, alveolar type 1; AT2, alveolar type 2; PDPN, Podoplanin; SARS-CoV-2, Severe Acute Respiratory Syndrome Coronavirus 2. (TIF) [file pbio.3001989.s003.tif]

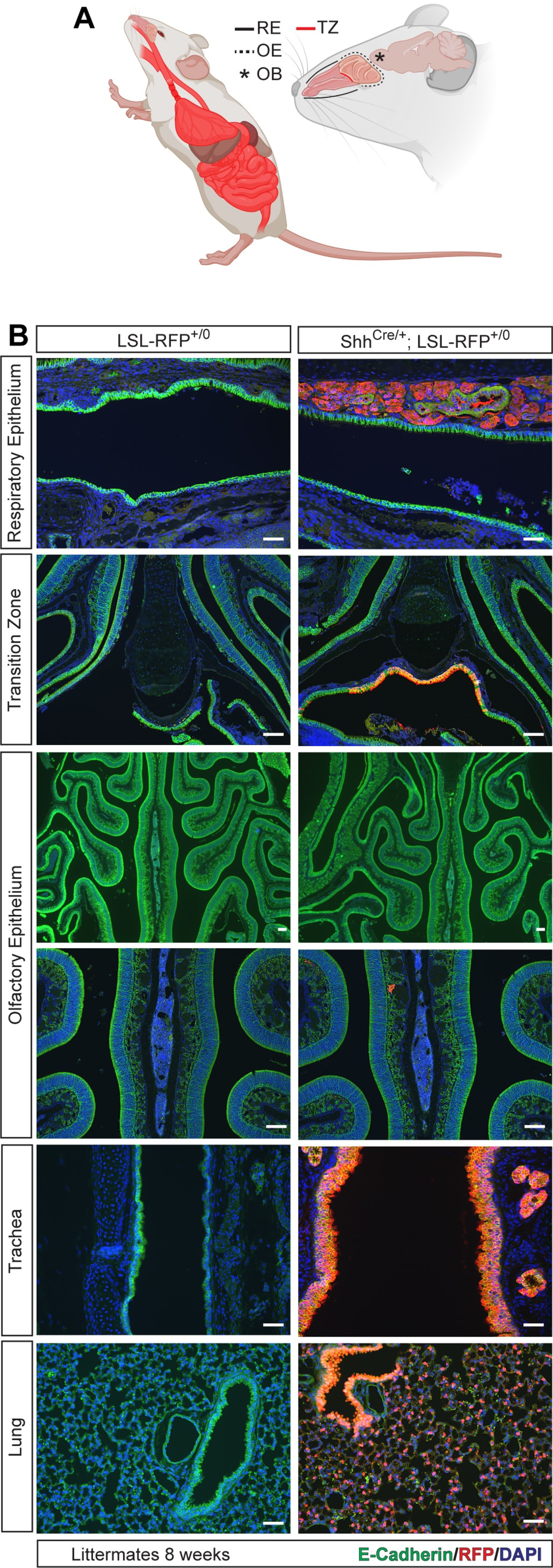

Supplement: S4 Fig — (A) Sites of ShhCre activity in respiratory and gut epithelium are shown in red. * indicates OB. Lines trace regions of nasal cavity epithelium. (B) Lineage trace of ShhCre activity in upper and lower RE using a Cre-activated, tdTomato allele (LSL-RFP). Immunohistochemistry using E-cadherin (epithelium) and RFP (Cre reporter) antibodies is shown. N = 3 per genotype. Scale bars 100 μm. Note: The LSL-RFP+/0 data (left) in panel B are the same in S11A Fig. While these animals were not littermates, the respective tissue was sectioned and immunostained contemporaneously on the same slide for stringent comparison. OB, olfactory bulb; OE, olfactory epithelium; RE, respiratory epithelium; RFP, Red Fluorescent Protein; TZ, transition zone. (TIF) [file pbio.3001989.s004.tif]

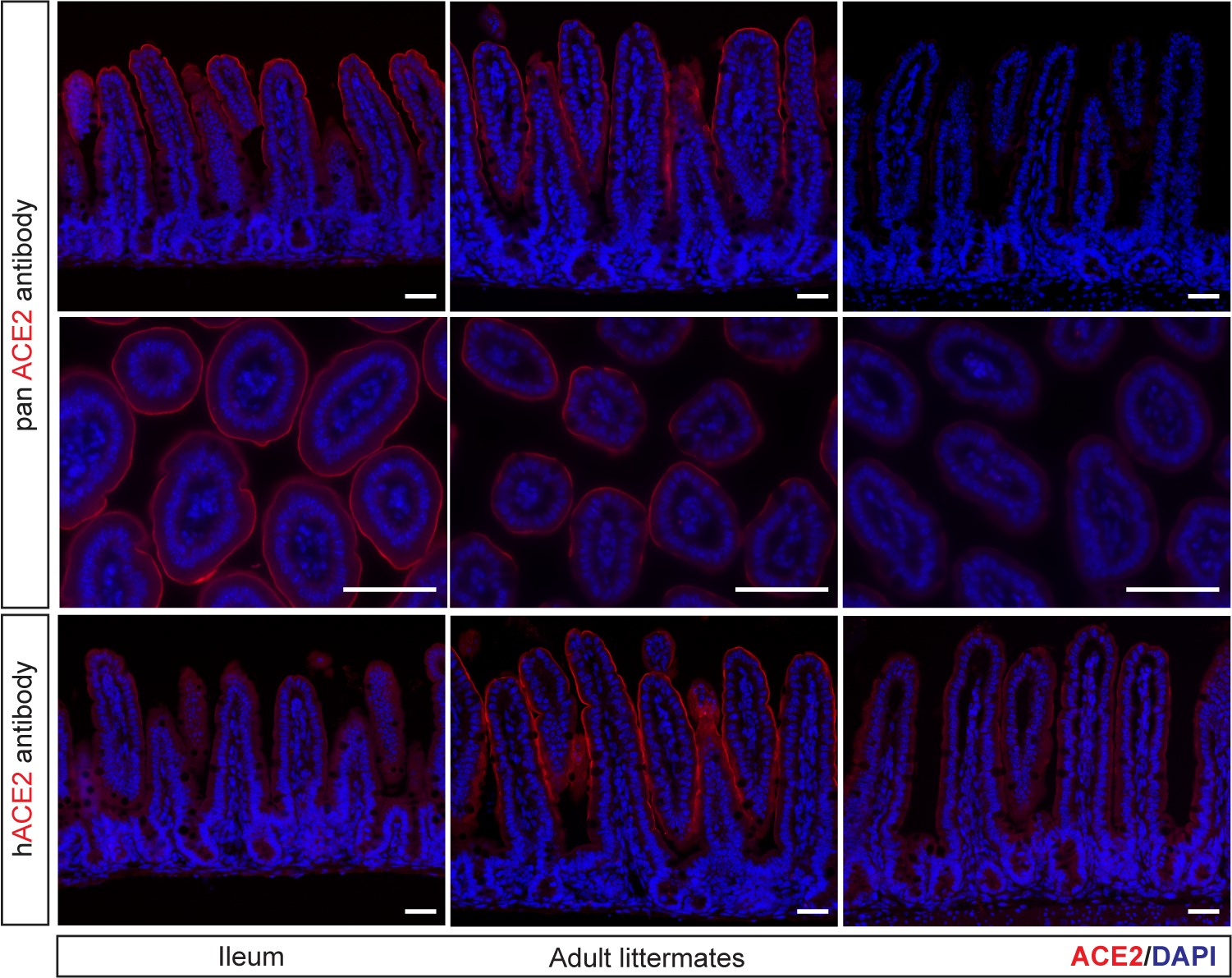

Supplement: S5 Fig — Immunohistochemistry of wild-type, hACE2fl/y, and ShhCre; hACE2fl/y small intestine using antibodies that recognize both human and mouse ACE2 (pan-ACE2) and only hACE2 are shown. Scale bars 50 μm. Representative of n = 3 per genotype and 2 independent litters. hACE2, human ACE2. (TIF) [file pbio.3001989.s005.tif]

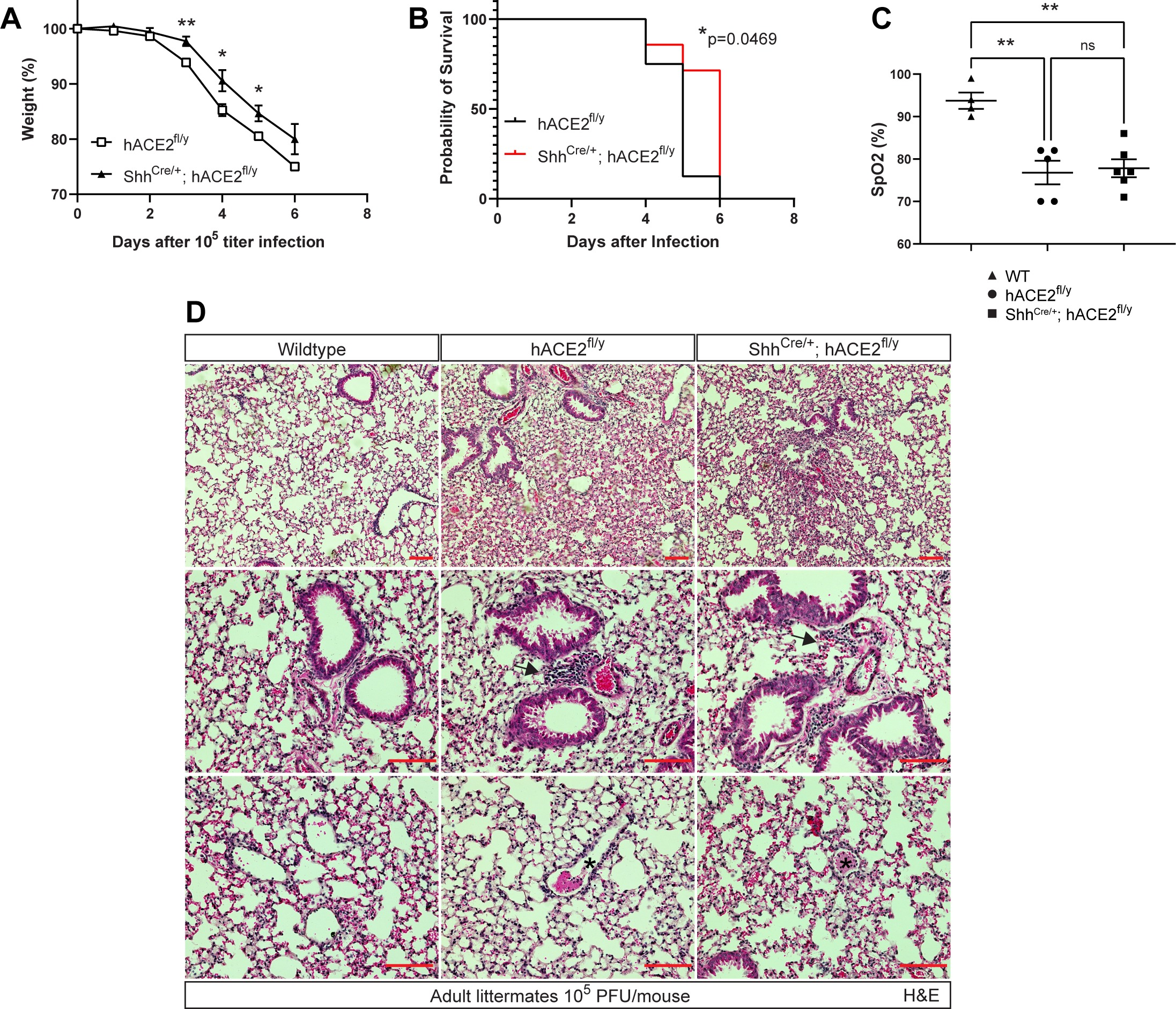

Supplement: S6 Fig — (A, B) Weight loss and survival of hACE2fl/y and ShhCre/+; hACE2fl/y mice after infection with 105 PFU of SARS-CoV-2. N = 8 (hACE2fl/y) and 7 (ShhCre/+; hACE2fl/y), two independent experiments. Note: Data for hACE2fl/y are the same as those shown in Fig 2D and 2E because ShhCre/+; hACE2fl/y animals were littermates of those animals. (C) Pulse oximetry measured in WT, hACE2fl/y, and ShhCre/+; hACE2fl/y mice at time of harvest 5–6 days after exposure to 105 PFU of SARS-CoV-2 virus. (D) HE staining of WT, hACE2fl/y, and ShhCre/+; hACE2fl/y lung tissue 6 days after exposure to 105 PFU of SARS-CoV-2 virus. Arrows, sites of bronchovascular immune cell infiltrate. Asterisks, intravascular thrombus. Representative of N = 3 animals per genotype. Scale bars 100 μm. Note: The hACE2fl/y data shown in panels A and B are the same as those shown in Fig 2, panels B-G. *p < 0.05, **p < 0.01, ns p > 0.05 by unpaired two-tailed t test, one-way ANOVA with Holm–Sidak correction for multiple comparisons, or log-rank Mantel Cox test. These hACE2fl/y mice were littermates of the ShhCre/+; hACE2fl/y mice and contemporaneously infected. Numerical data in corresponding S1 Metadata tab. HE, hematoxylin–eosin; PFU, plaque-forming unit; SARS-CoV-2, Severe Acute Respiratory Syndrome Coronavirus 2; WT, wild-type. (TIF) [file pbio.3001989.s006.tif]

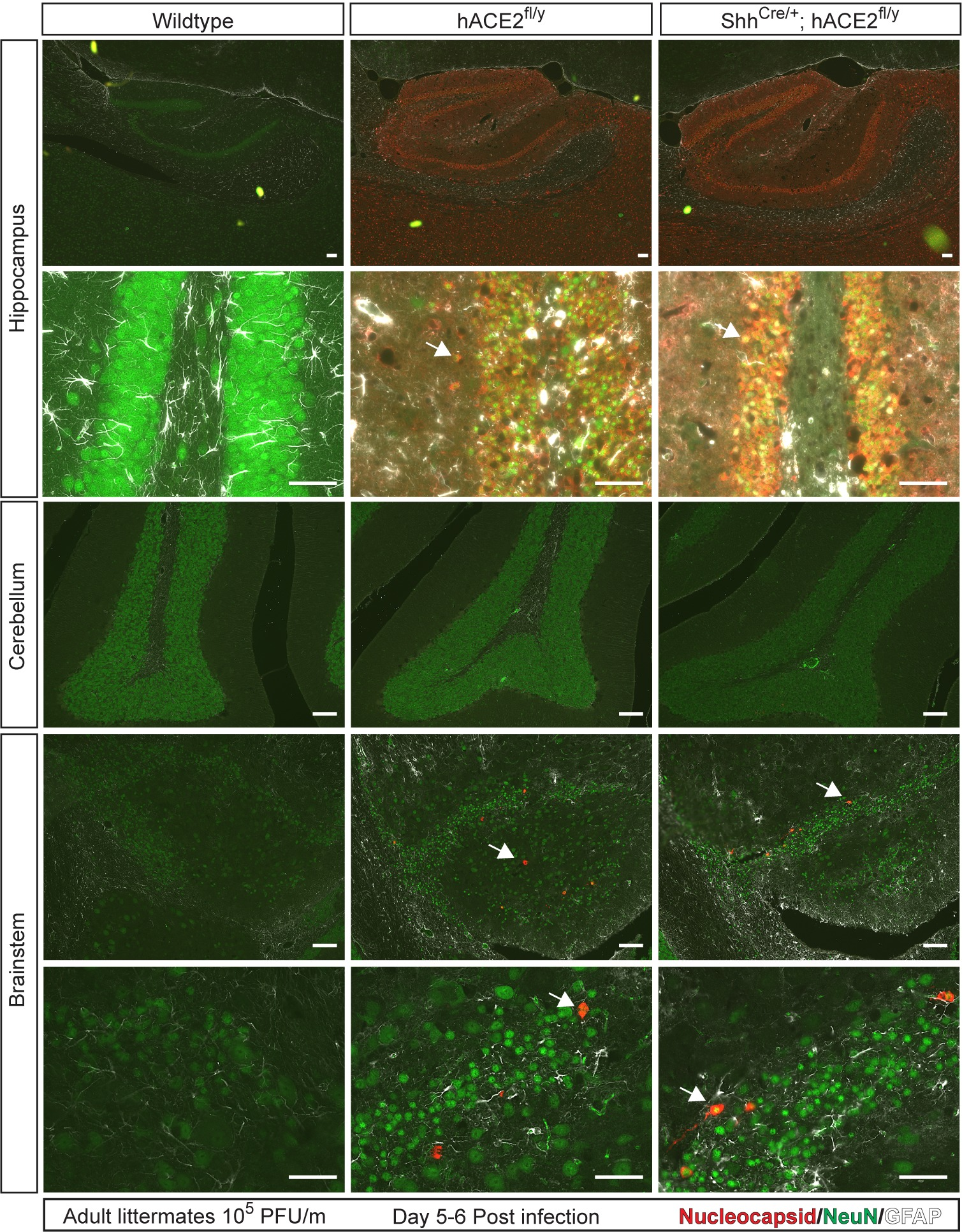

Supplement: S7 Fig — Immunohistochemistry of wild-type, hACE2fl/y, and ShhCre/+; hACE2fl/y hippocampus, cerebellum, and brainstem with antibody staining for viral nucleocapsid, neurons (NeuN), and glial cells (GFAP) is shown 5–6 days after infection with SARS-CoV-2. Arrows indicate NeuN-positive neurons with colocalized nucleocapsid staining. N = 3 per genotype. Scale bars 100 μm (top three rows), 50 μm (bottom two rows). SARS-CoV-2, Severe Acute Respiratory Syndrome Coronavirus 2. (TIF) [file pbio.3001989.s007.tif]

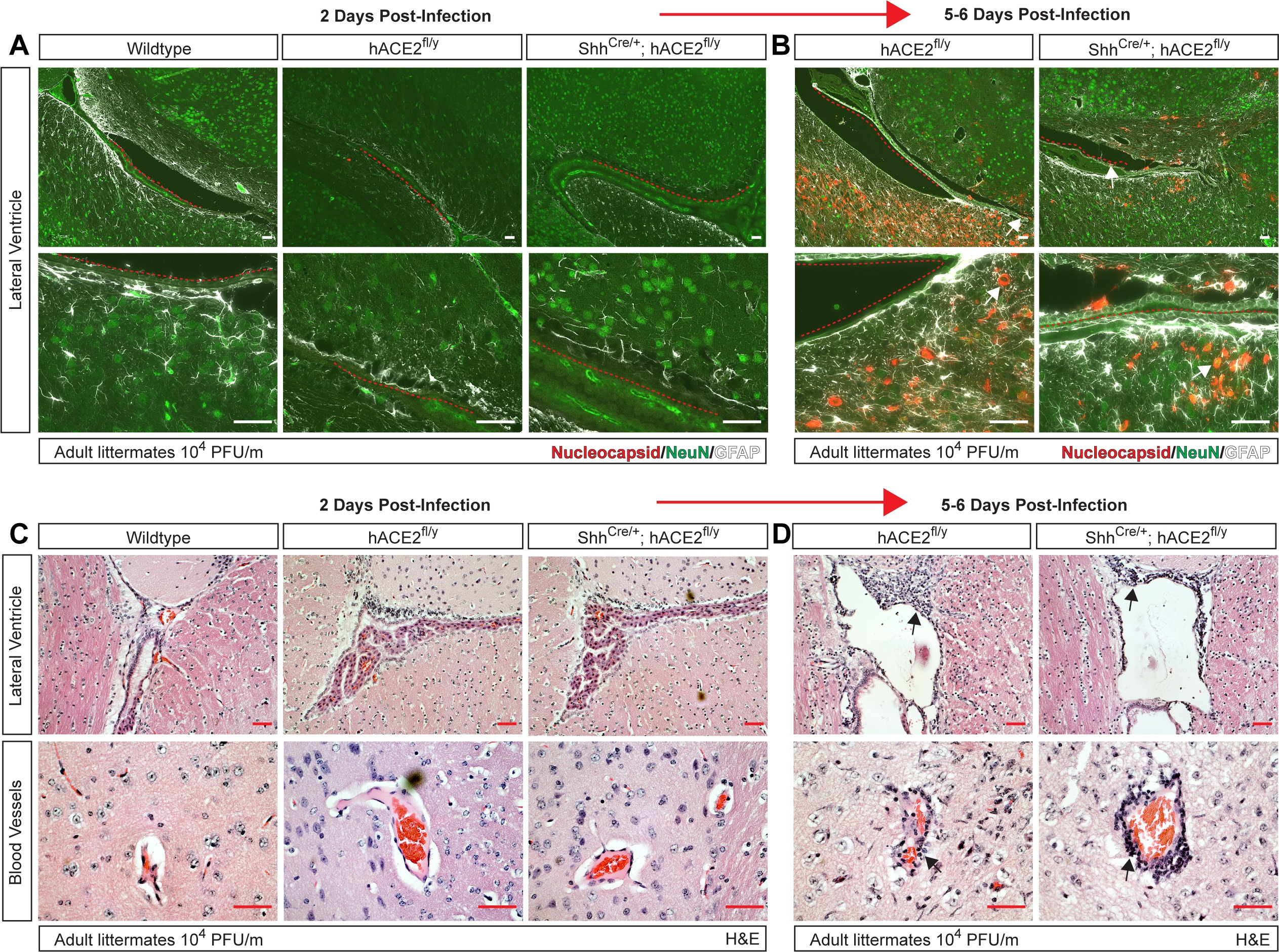

Supplement: S8 Fig — (A, B) Immunohistochemistry of SARS-CoV-2 nucleocapsid, neuronal NeuN, and glial cell GFAP of the cerebral cortex adjacent to the choroid plexus 2 and 5–6 days after infection. Arrows indicated nucleocapsid staining. Red dotted lines trace ependymal cells of the choroid plexus. (C, D) HE staining of brain choroid plexus and cerebral cortex blood vessels 2 and 5–6 days after infection of hACE2fl/y and ShhCre/+; hACE2fl/y mice. Arrows indicate sites of immune cell infiltration. Representative of N = 4 animals per genotype and time point. Scale bars 50 μm. HE, hematoxylin–eosin; SARS-CoV-2, Severe Acute Respiratory Syndrome Coronavirus 2. (TIF) [file pbio.3001989.s008.tif]

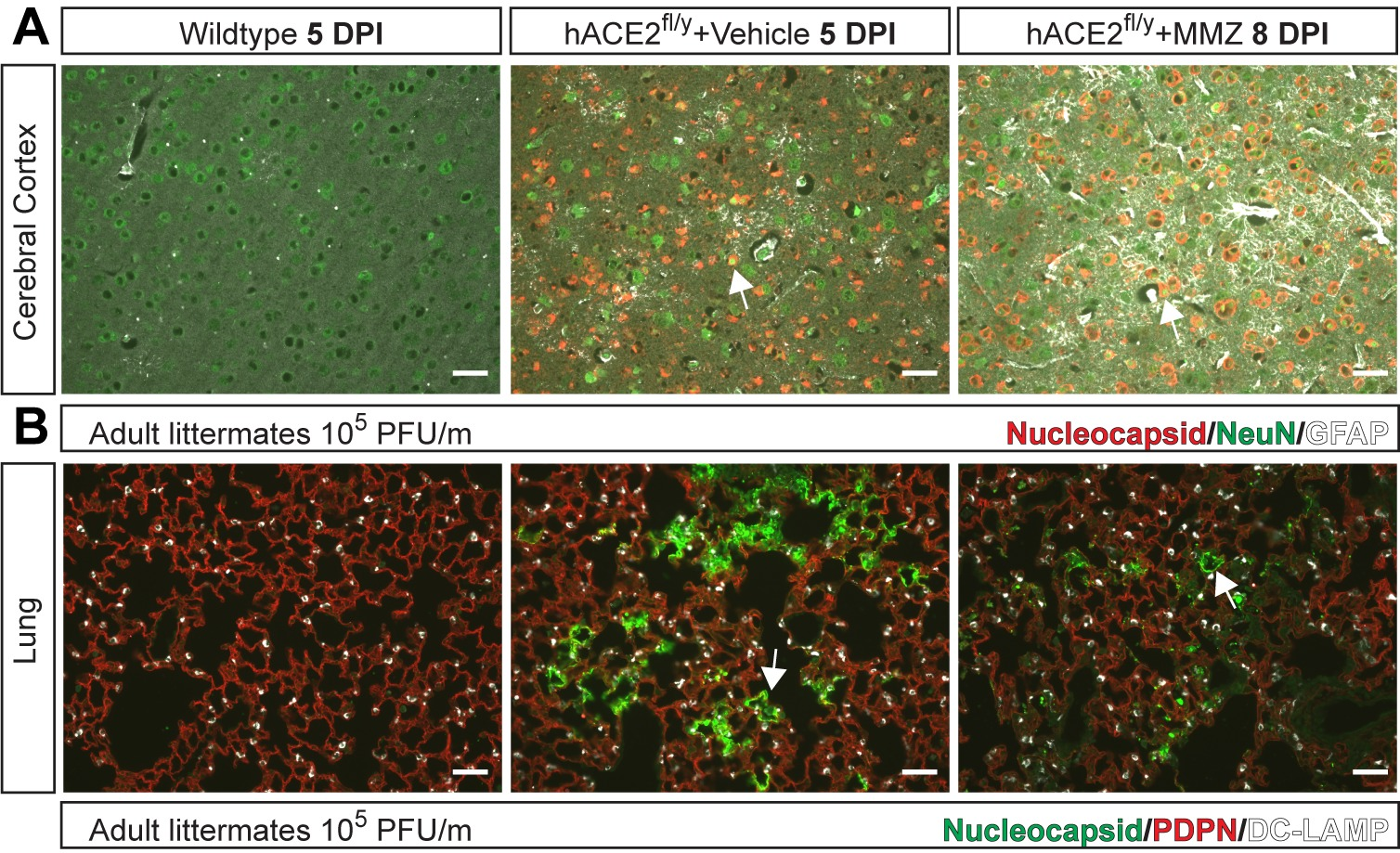

Supplement: S9 Fig — (A, B) Cerebral cortex (A) and lung (B) immunohistochemistry of hACE2fl/y animals treated with vehicle or MMZ followed by infection with 105 viral titer. The tissues shown on the far right (“hACE2fl + MMZ 8 DPI”) are from a hACE2fl mouse that exhibited weight loss and required euthanasia 8 days following infection with 105 SARS-CoV-2 despite pretreatment with MMZ. Cerebral cortex stained with viral nucleocapsid, neuronal NeuN, and glial cell GFAP. Lung stained with viral nucleocapsid, AT1 cells PDPN, and AT2 cell DC-LAMP. Arrows indicate nucleocapsid staining colocalized with NeuN+ neurons or PDPN+ AT1 cells. N = 4 per genotype except for N = 1 (MMZ treated). Scale bars 50 μm. AT1, alveolar type 1; AT2, alveolar type 2; MMZ, methimazole; SARS-CoV-2, Severe Acute Respiratory Syndrome Coronavirus 2. (TIF) [file pbio.3001989.s009.tif]

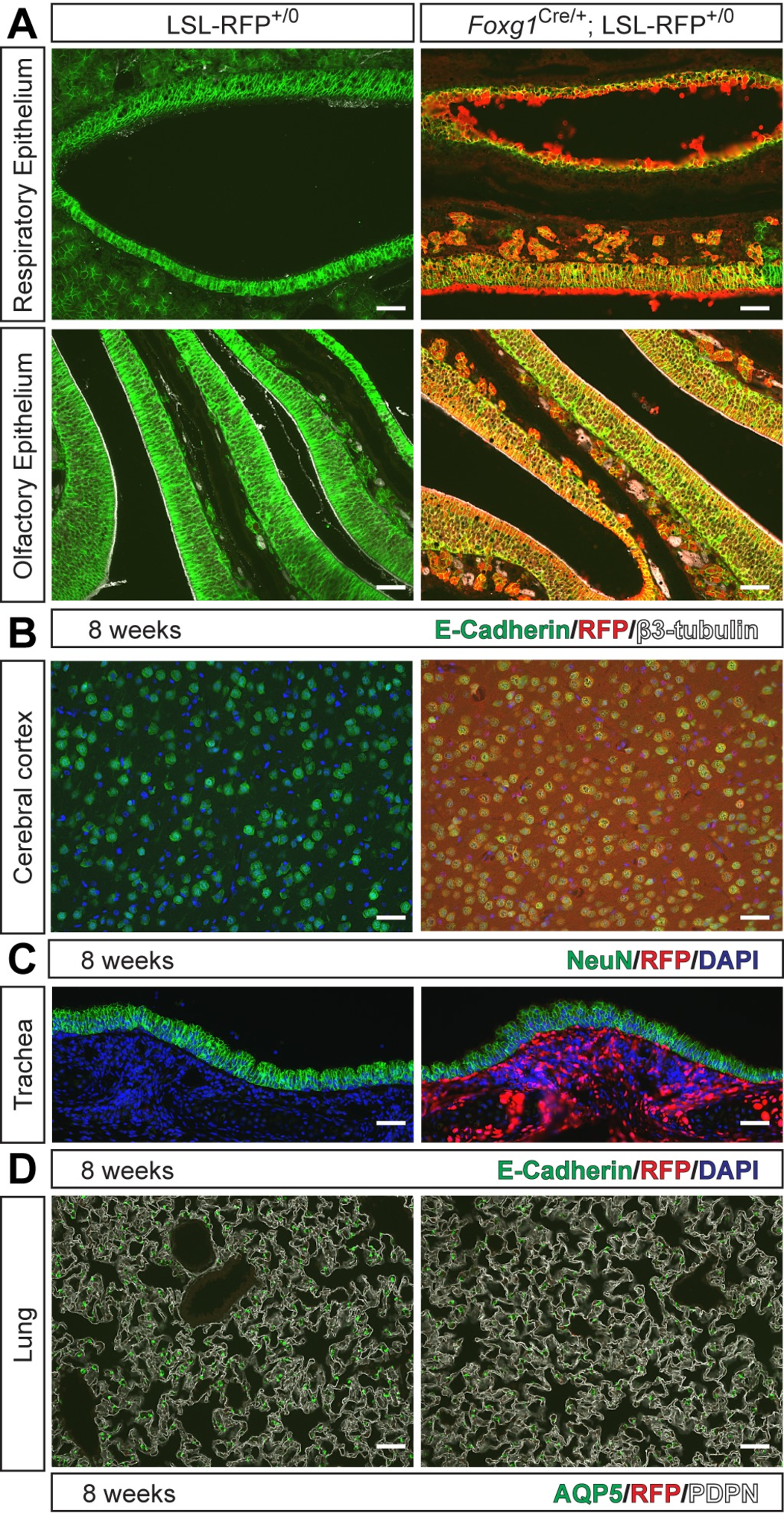

Supplement: S10 Fig — Foxg1Cre was crossed to a Cre-activated, tdTomato allele (LSL-RFP) to trace activity along the route of SARS-CoV-2 infection. Sites of Foxg1Cre activity are shown in red. (A, B) Immunostaining to detect E-cadherin, NeuN, and RFP demonstrate that Foxg1Cre is active in RE, OE, and neurons in the brain. (B) Immunostaining to detect E-cadherin, AQP5, PDPN, and RFP demonstrate that Foxg1Cre is not active in tracheal or lung epithelial cells. Cre activity was detected in an E-cadherin negative, sub-epithelial population of cells in the trachea I. N = 3 per genotype. Scale bars 50 μm. AQP5, Aquaporin 5; OE, olfactory epithelium; PDPN, Podoplanin; RE, respiratory epithelium; RFP, Red Fluorescent Protein; SARS-CoV-2, Severe Acute Respiratory Syndrome Coronavirus 2. (TIF) [file pbio.3001989.s010.tif]

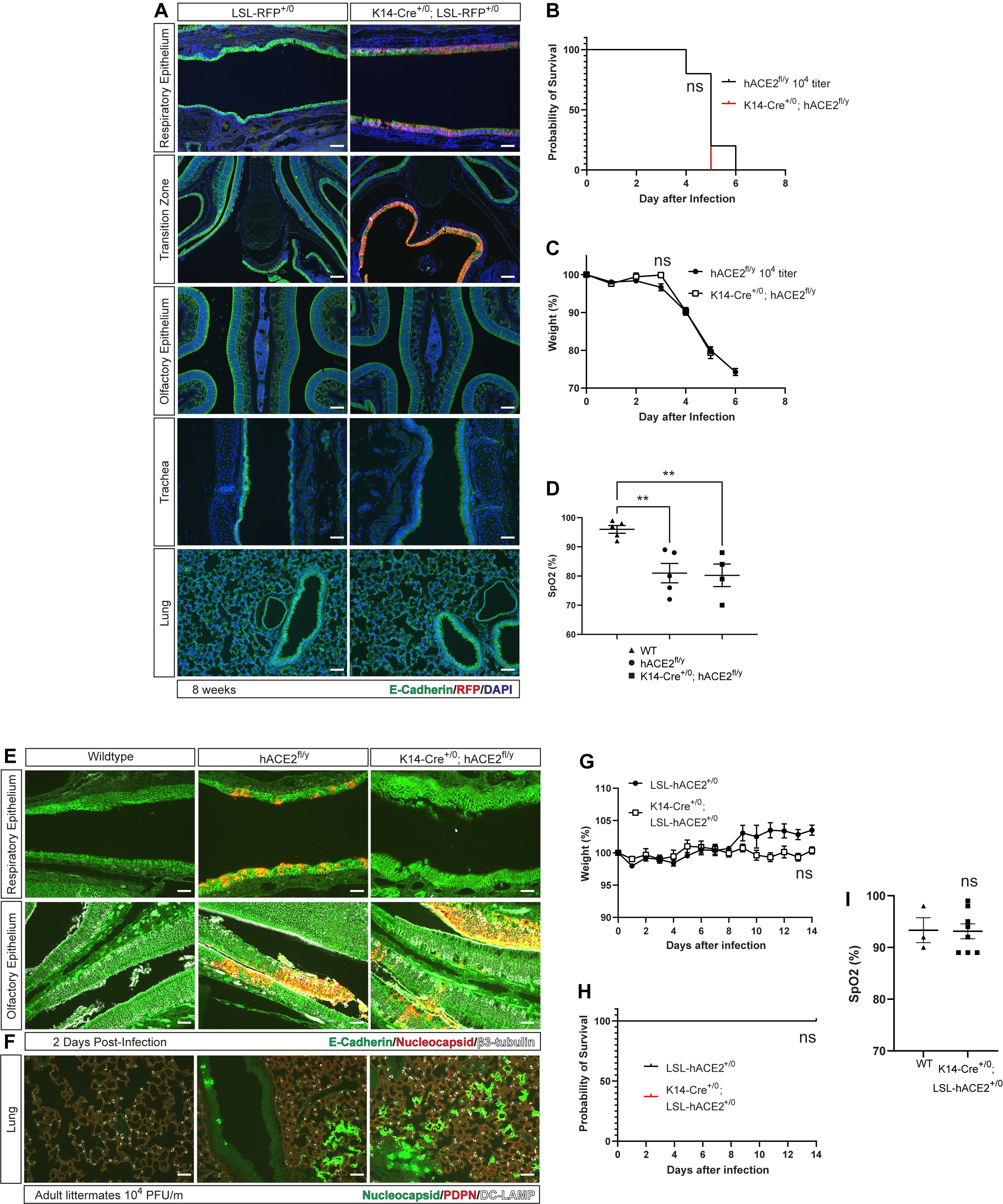

Supplement: S11 Fig — (A) A K14-Cre transgene was crossed to a Cre-activated, tdTomato allele (LSL-RFP) to trace activity along the route of SARS-CoV-2 infection. Immunostaining for pan-epithelial cell marker E-cadherin and Cre reporter RFP was performed in nasal cavity, trachea and lung. n = 3. Scale bars 100 μm. (B, C) Survival and weight loss of hACE2fl/y and K14-Cre; hACE2fl/y mice after infection with 104 PFU of SARS-CoV-2. N = 5 (hACE2fl/y) and 5 (K14-Cre; hACE2fl/y), one experiment. (D) Pulse oximetry measured in WT, hACE2fl/y, and K14-Cre; hACE2fl/y mice 6 days after exposure to 104 PFU of SARS-CoV-2. (E, F) Immunohistochemistry of WT, hACE2fl/y, and K14-Cre; hACE2fl/y RE, OE, and lung tissue 2 days after exposure to 104 PFU of SARS-CoV-2 was performed using antibodies against E-cadherin (epithelial cells), SARS-CoV-2 nucleocapsid, β3-tubulin (neurons), DC-LAMP (alveolar type 2 cells), and PDPN (alveolar type 1 cells). Note the loss of viral infection of RE but not OE or lung epithelium in K14-Cre; hACE2fl/y mice. Representative of N = 4 animals per genotype. Scale bars, 50 μm. (G, H) Weight loss and survival of LSL-hACE2+/0 and K14-Cre; LSL-hACE2+/0 mice after infection with 104 PFU of SARS-CoV-2. N = 3 (LSL-hACE2+/0) and 8(K14-Cre; LSL-hACE2+/0). (I) Pulse oximetry of WT (LSL-hACE2+/0 or K14-Cre;Ace2+/+) and K14-Cre; LSL-hACE2+/0 mice infected with 104 viral titer 6 days after infection. Note: The LSL-RFP+/0 data (left) in panel A are the same in S4B Fig. While these animals were not littermates, the respective tissue was sectioned and immunostained contemporaneously on the same slide for stringent comparison. ns, not significant p > 0.05. **p < 0.01 by unpaired two-tailed t test, one-way ANOVA with Holm–Sidak correction for multiple comparisons, or log-rank Mantel Cox test. Numerical data in corresponding S1 Metadata tab. hACE2, human ACE2; OE, olfactory epithelium; PFU, plaque-forming unit; RE, respiratory epithelium; RFP, Red Fluorescent Protein; SARS-CoV-2, Severe Acute Res [file pbio.3001989.s011.tif]

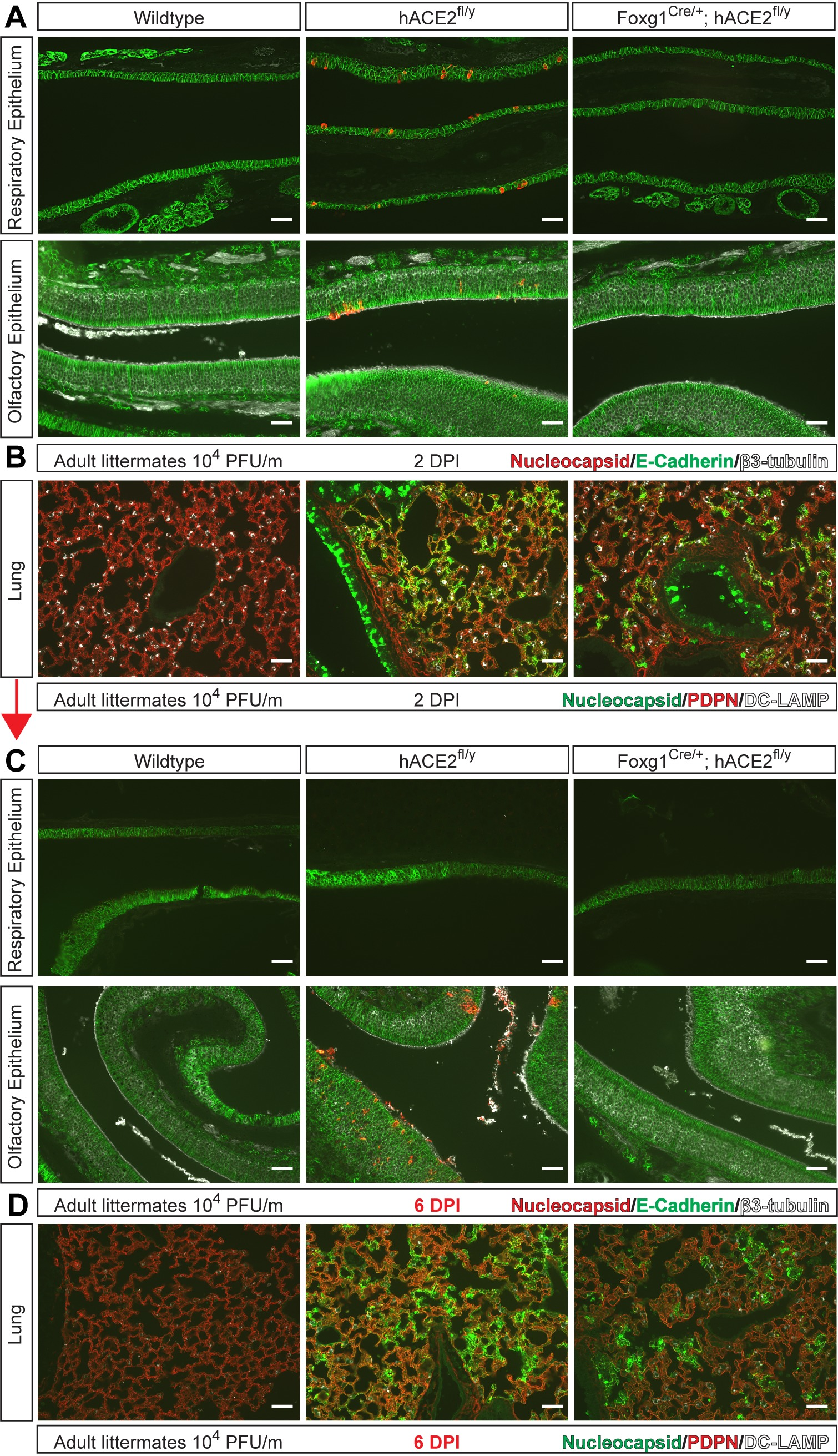

Supplement: S12 Fig — (A, B) Immunohistochemistry of SARS-CoV-2 nucleocapsid, epithelial E-cadherin, neuronal β3 tubulin, AT1 cell PDPN, and AT2 cell DC-LAMP in the respiratory, olfactory, and pulmonary epithelium 2 days after infection of wild-type, hACE2fl/y, and Foxg1Cre; hACE2fl/y mice. Representative of N = 3 animals per genotype and time point. Scale bars 50 μm. (C, D) Immunohistochemistry of SARS-CoV-2 nucleocapsid, epithelial E-cadherin, neuronal β3 tubulin, AT1 cell PDPN, and AT2 cell DC-LAMP in the respiratory, olfactory, and pulmonary epithelium 6 days after infection of wild-type, hACE2fl/y, and Foxg1Cre; hACE2fl/y mice. Representative of N = 4 animals per genotype and time point. Scale bars 50 μm. AT1, alveolar type 1; AT2, alveolar type 2; hACE2, human ACE2; SARS-CoV-2, Severe Acute Respiratory Syndrome Coronavirus 2. (TIF) [file pbio.3001989.s012.tif]

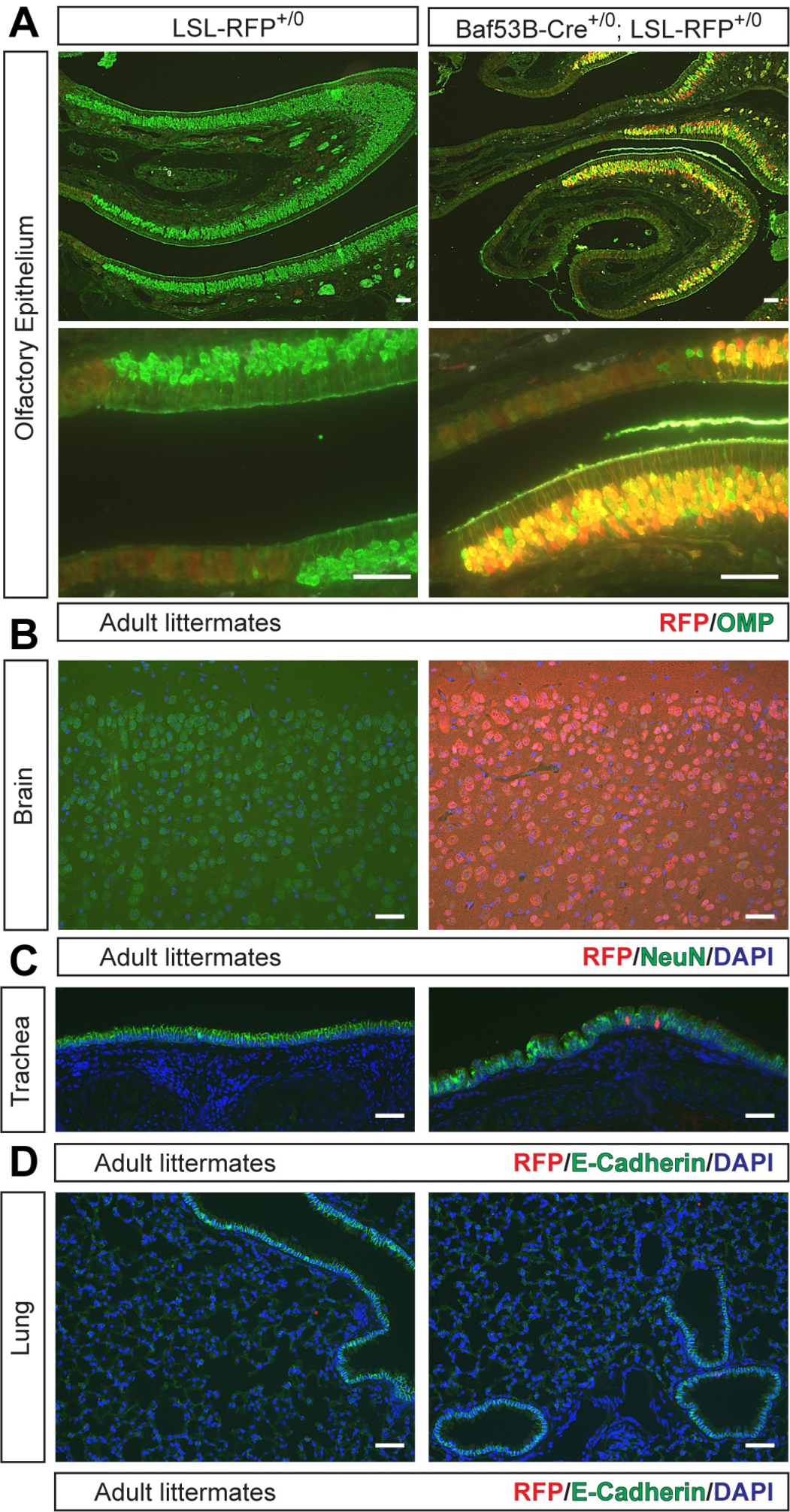

Supplement: S13 Fig — Lineage tracing of Baf53b-Cre activity along the route of SARS-CoV-2 infection was performed using a Cre-activated, tdTomato allele (LSL-RFP). (A) Immunostaining for RFP and OMP, a marker of OSNs, in OE LSL-hACE2+/0 and Baf53b-Cre; LSL-hACE2+/0 mice. (B) Immunostaining for RFP and NeuN, a neuronal marker, in the brain of LSL-hACE2+/0 and Baf53b-Cre; LSL-hACE2+/0 mice. (C, D) Immunostaining for RFP and E-cadherin, an epithelial cell marker, in the trachea and lung of LSL-hACE2+/0 and Baf53b-Cre; LSL-hACE2+/0 mice. N = 3 per genotype. Scale bars in all images 50 μm. OE, olfactory epithelium; OMP, olfactory marker protein; OSN, olfactory sensory neuron; RFP, Red Fluorescent Protein; SARS-CoV-2, Severe Acute Respiratory Syndrome Coronavirus 2. (TIF) [file pbio.3001989.s013.tif]

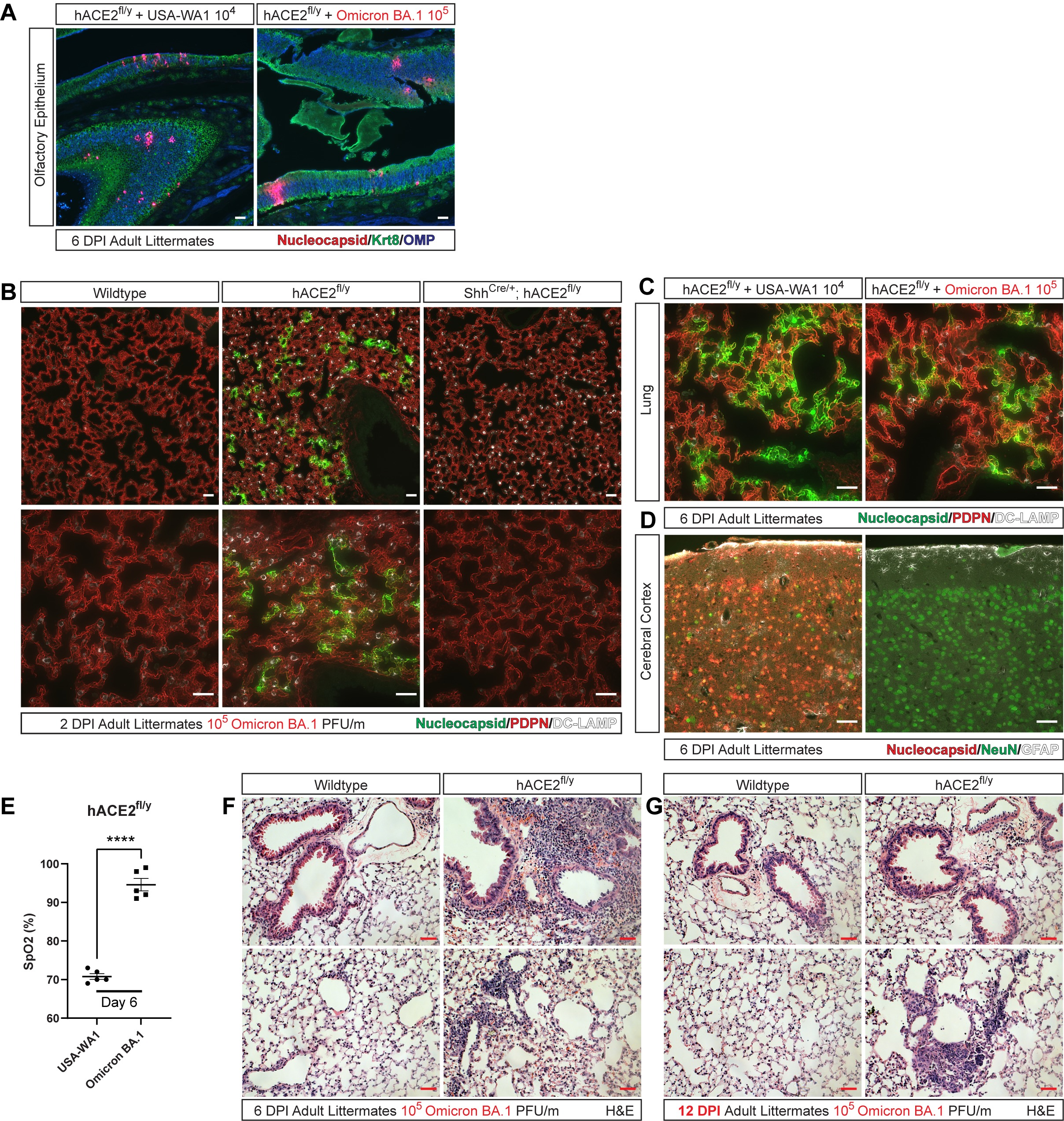

Supplement: S14 Fig — (A) Immunostaining for SARS-CoV-2 nucleocapsid in the OE of hACE2fl/y mice 6 days after infection with the indicated doses of USA-WA1 or Omicron BA.1 variants. (B) Immunohistochemistry of wild-type, hACE2fl/y, and ShhCre; hACE2fl/y lung 2 DPI with 105 PFU of Omicron BA.1 SARS-CoV-2 virus was performed using antibodies that recognize viral nucleocapsid, AT1 cell PDPN, and AT2 cell DC-LAMP. N = 4 per genotype. (C) Immunohistochemistry of hACE2fl/y lung 6 DPI with 104 PFU of USA-WA1 or 105 PFU of Omicron BA.1 SARS-CoV-2 virus was performed using antibodies that recognize viral nucleocapsid, AT1 cell PDPN, and AT2 cell DC-LAMP. N = 5 per genotype. (D) Immunohistochemistry of hACE2fl/y lung 6 DPI with 104 PFU of USA-WA1 or 105 PFU of Omicron BA.1 SARS-CoV-2 virus was performed using antibodies that recognize viral nucleocapsid, neuronal NeuN, and glial cell GFAP. N = 5 per genotype. (E) Pulse oximetry of hACE2fl/y mice infected with 104 PFU of USA-WA1 or 105 PFU of Omicron BA.1 SARS-CoV-2 virus 6 DPI. ****p < 0.0001 determined by unpaired, two-tailed t test. (F) HE staining of wild-type and hACE2fl/y lung tissue 6 (F) and 12 (G) days after exposure to 105 PFU of SARS-CoV-2 virus. Representative of n = 3 animals per genotype. Scale bars in all images 50 μm. Numerical data in corresponding S1 Metadata tab. AT1, alveolar type 1; AT2, alveolar type 2; DPI, days postinfection; OE, olfactory epithelium; PDPN, Podoplanin; PFU, plaque-forming unit; SARS-CoV-2, Severe Acute Respiratory Syndrome Coronavirus 2. (TIF) [file pbio.3001989.s014.tif]

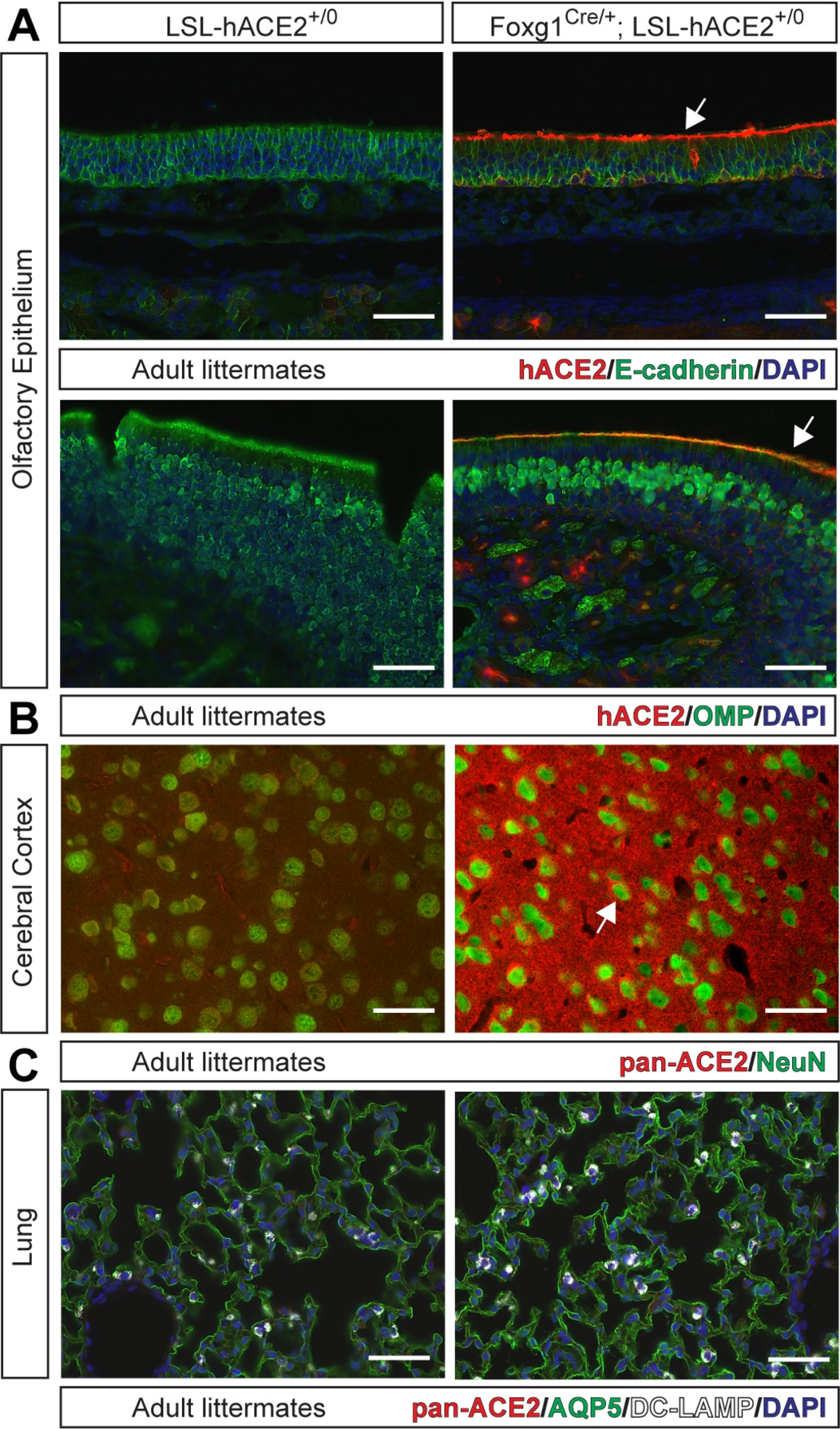

Supplement: S15 Fig — (A-C) Expression of hACE2 was detected using either anti-hACE2 or anti-panACE2 antibodies in Foxg1Cre;LSL-hACE2+/0 and LSL-hACE2+/0 littermate control mice. Costaining for E-cadherin, OMP, NeuN, AQP5, and CD-LAMP was used to label epithelial cells, OSNs, neurons, AT1, and AT2 cells, respectively. Arrows indicate sites of hACE2 expression in OE and cerebral cortex. N = 3 per genotype. Scale bars 50 μm. AQP5, Aquaporin 5; AT1, alveolar type 2; AT2, alveolar type 2; hACE2, human ACE2; OE, olfactory epithelium; OMP, olfactory marker protein; OSN, olfactory sensory neuron. (TIF) [file pbio.3001989.s015.tif]

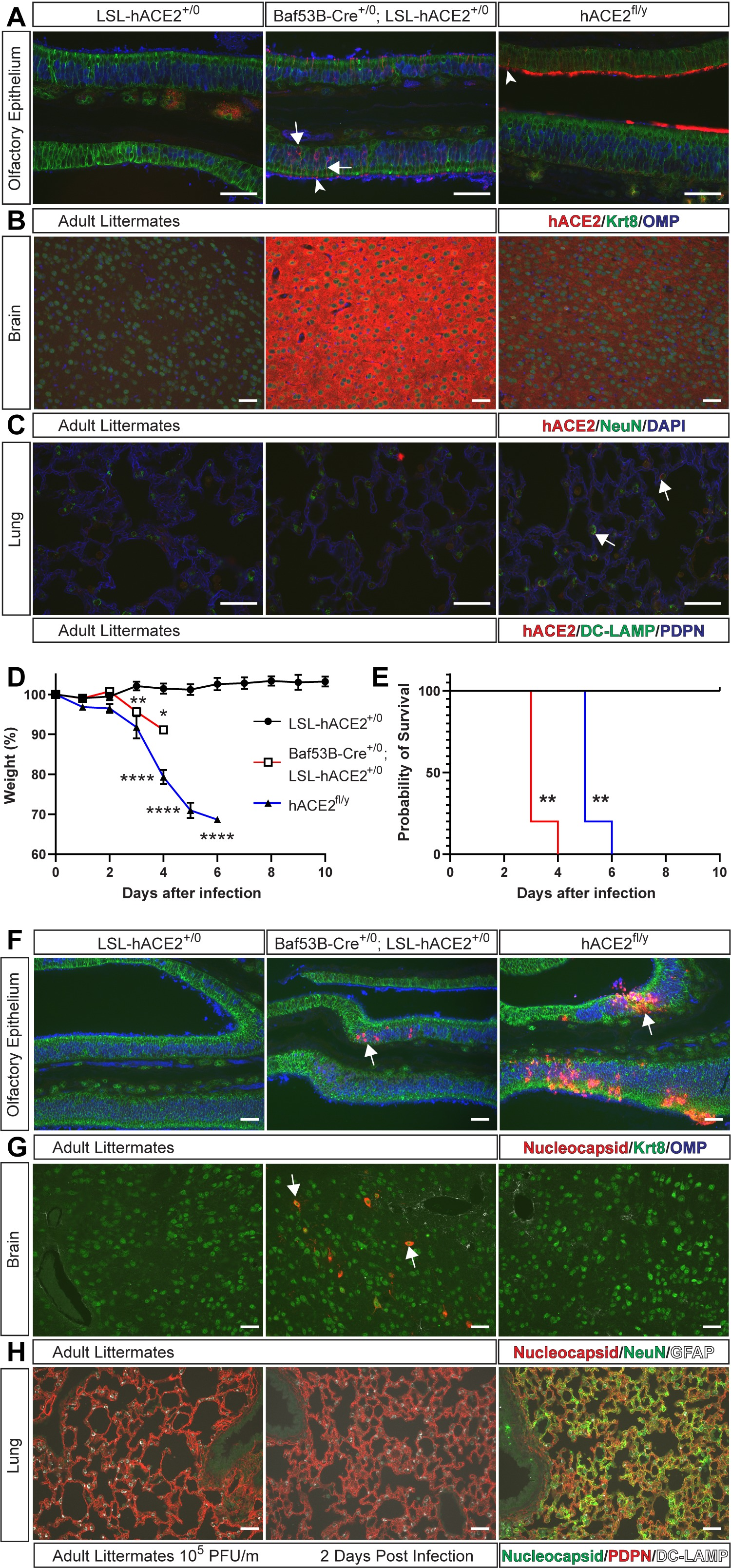

Supplement: S16 Fig — (A-C) Immunohistochemistry of LSL-hACE2+/0, Baf53b-Cre+/0;LSL-hACE2+/0, and hACE2fl/y mouse OE (A), brain (B), and lung (C) using antibodies that recognize hACE2 and markers of OE (Krt8 and OMP), neurons (NeuN), and lung epithelial cells (DC-LAMP and PDPN). Arrows indicate OSNs and arrowheads indicate OSN cilia in (A). Arrows indicate faint expression of hACE2 in DC-LAMP+ AT2 cells in (C). Representative of n = 3 per genotype. (D, E) Weight loss and survival of LSL-hACE2+/0, Baf53b-Cre+/0;LSL-hACE2+/0, and hACE2fl/y mice after infection with 105 PFU of SARS-CoV-2. n = 5 for each genotype. Asterisks indicate time points at which significant differences in weight (D) or survival (E) were observed between Baf53b-Cre+/0;LSL-hACE2+/0 and hACE2fl/y animals compared to contemporaneously infected LSL-hACE2+/0 controls. (F-H) Immunohistochemistry of SARS-CoV-2 nucleocapsid and markers of OE (Krt8 and OMP), neurons (NeuN), glial cells (GFAP), and lung epithelial cells (DC-LAMP and PDPN) in the OE, brain, and lung 2 days after infection of LSL-hACE2+/0, Baf53b-Cre+/0;LSL-hACE2+/0, and hACE2fl/y mice. Arrows indicate sites of viral nucleocapsid detection. Representative of N = 3 animals per genotype and time point. Scale bars in all images 50 μm. *p < 0.05; **p < 0.01; ****p < 0.0001 determined by unpaired, two-tailed t test or log-rank Mantel Cox test. Numerical data in corresponding S1 Metadata tab. AT2, alveolar type 2; hACE2, human ACE2; OE, olfactory epithelium; OMP, olfactory marker protein; OSN, olfactory sensory neuron; PDPN, Podoplanin; PFU, plaque-forming unit; SARS-CoV-2, Severe Acute Respiratory Syndrome Coronavirus 2. (TIF) [file pbio.3001989.s016.tif]
