# Supplementary material for: Cell-autonomous requirement for ACE2 across organs in lethal mouse SARS-CoV-2 infection
Source: PLoS Biol. 2023 Feb 6;21(2):e3001989. doi: 10.1371/journal.pbio.3001989 (PMC9934376; doi:10.1371/journal.pbio.3001989)
Supplement: S1 Raw Images — In this file are unedited western blot images corresponding to those presented throughout the manuscript. The edited western blot is shown above the unedited blot. (PDF) [file pbio.3001989.s018.pdf]

Figure 1B

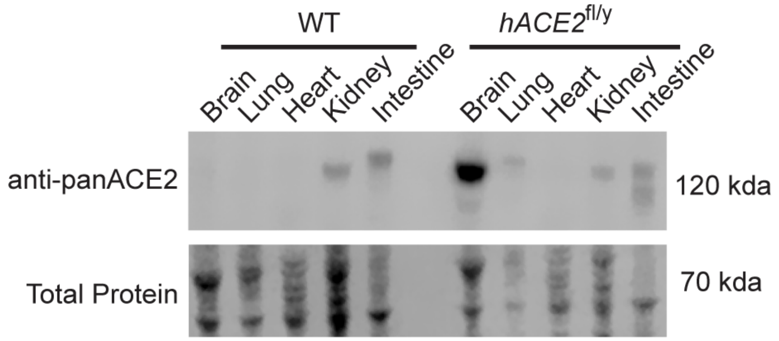

Anti-panACE2

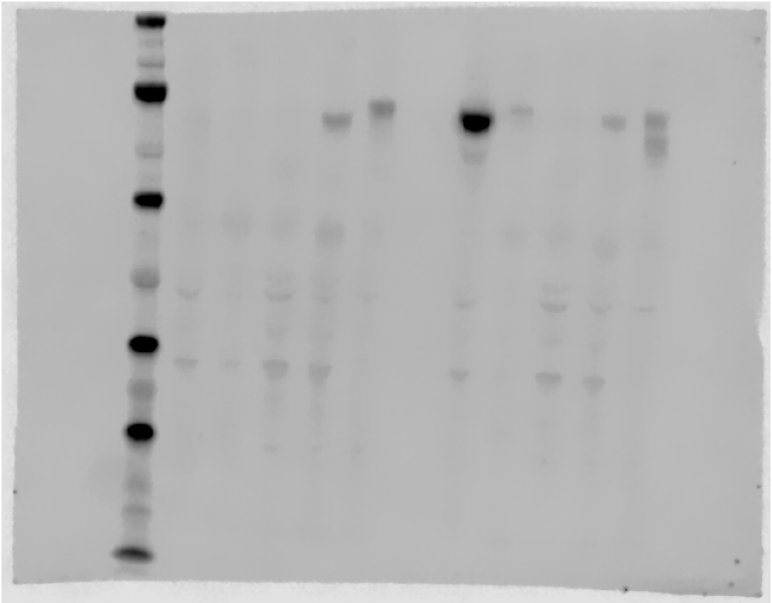

Total Protein

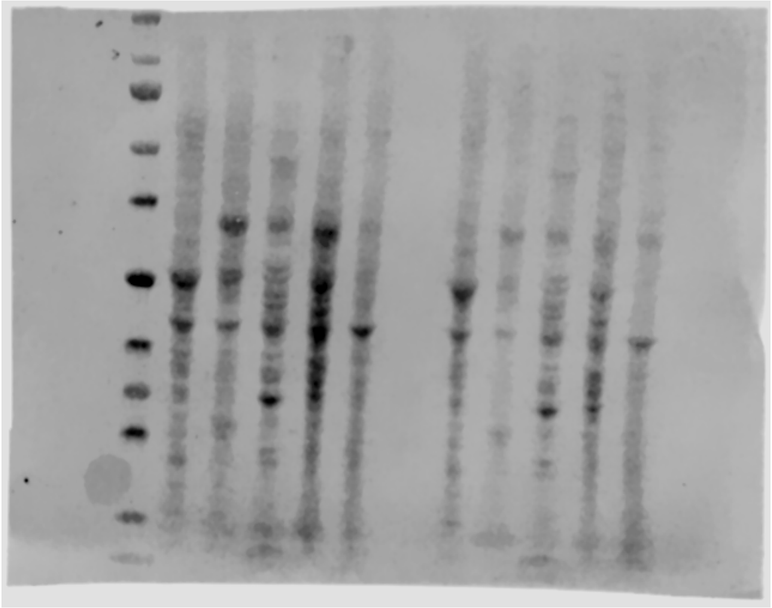

Figure 1D

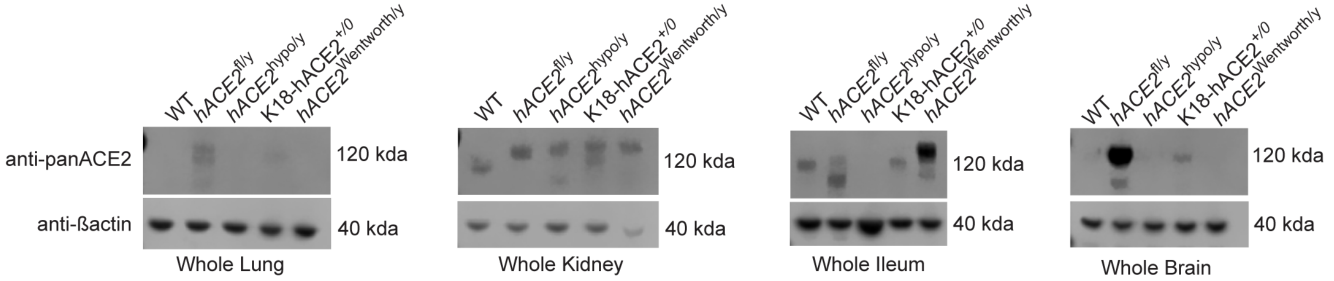
WT
hACE2fl/y
hACE2hypo/y
K18-hACE2+/0
hACE2Wentworth/y
anti-panACE2
120 kda
anti-ßactin
40 kda
Whole Lung
Whole Kidney
Whole Ileum
Whole Brain

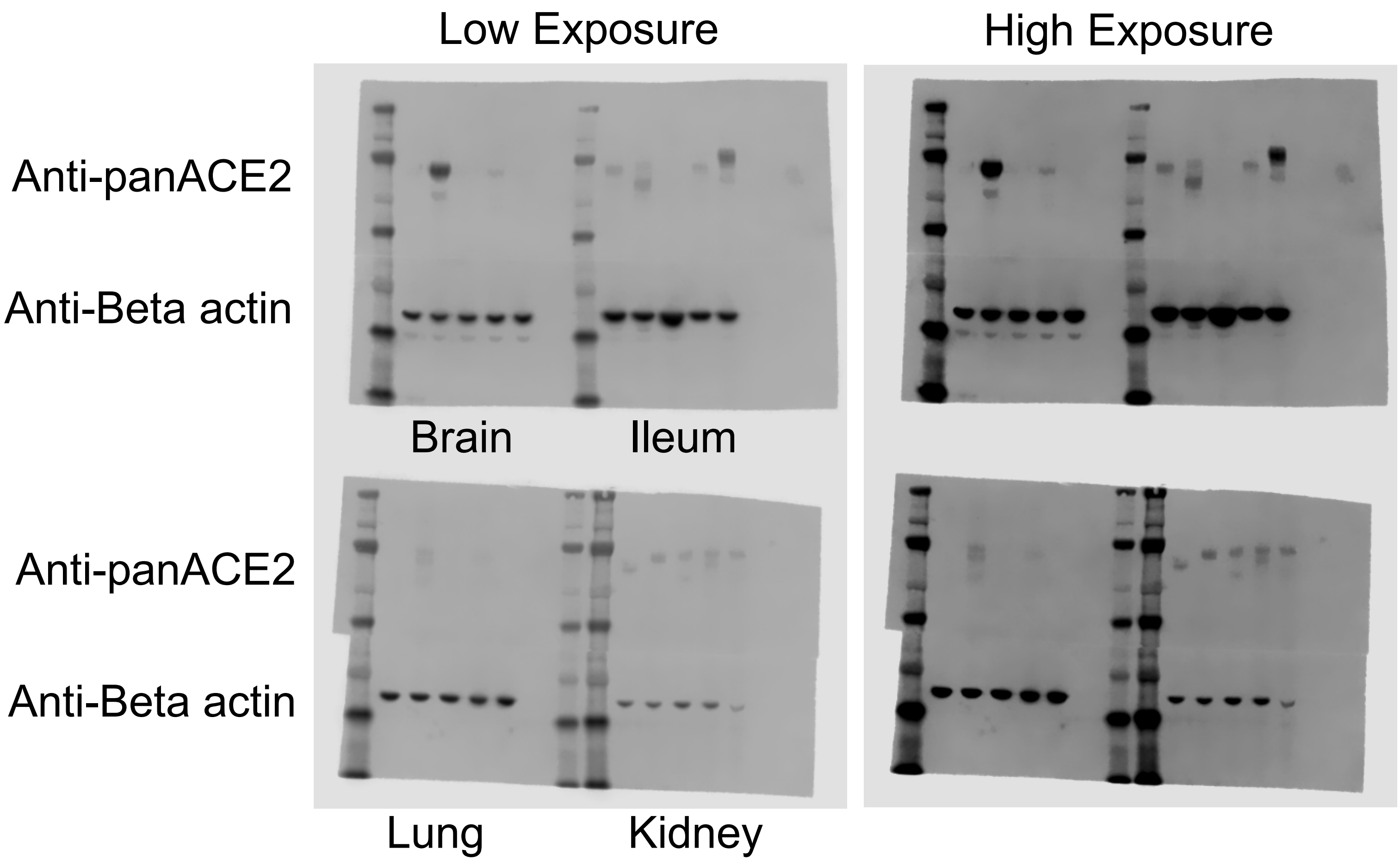
Low Exposure
High Exposure
Anti-panACE2
Anti-Beta actin
Brain
Ileum
Anti-panACE2
Anti-Beta actin
Lung
Kidney

Figure 1E

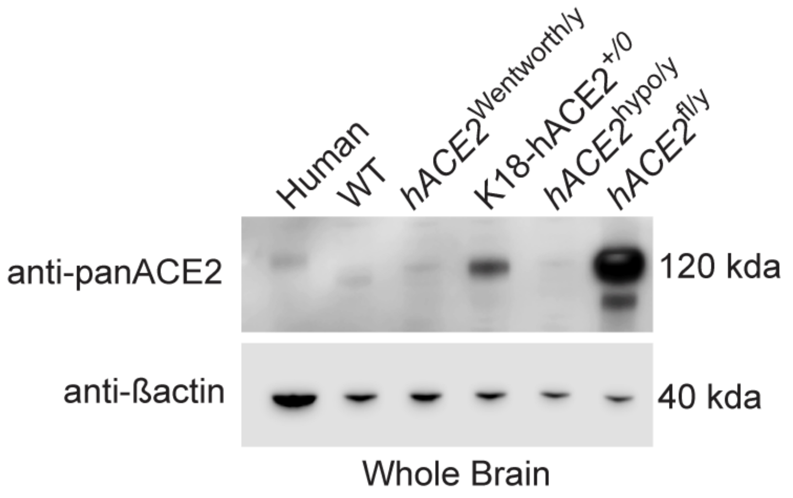

Low Exposure

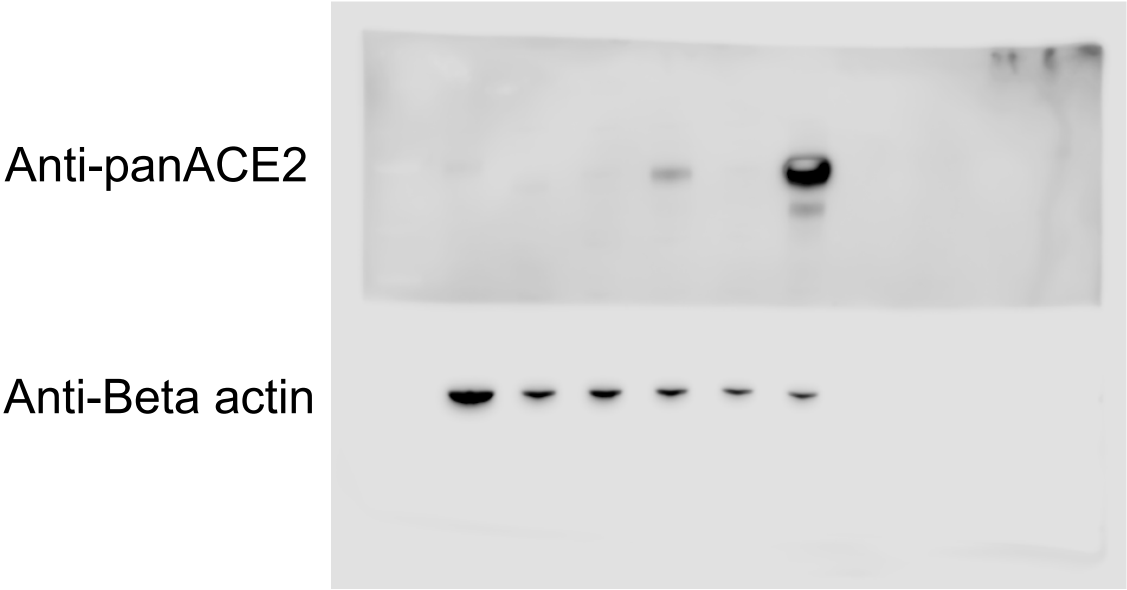

High Exposure

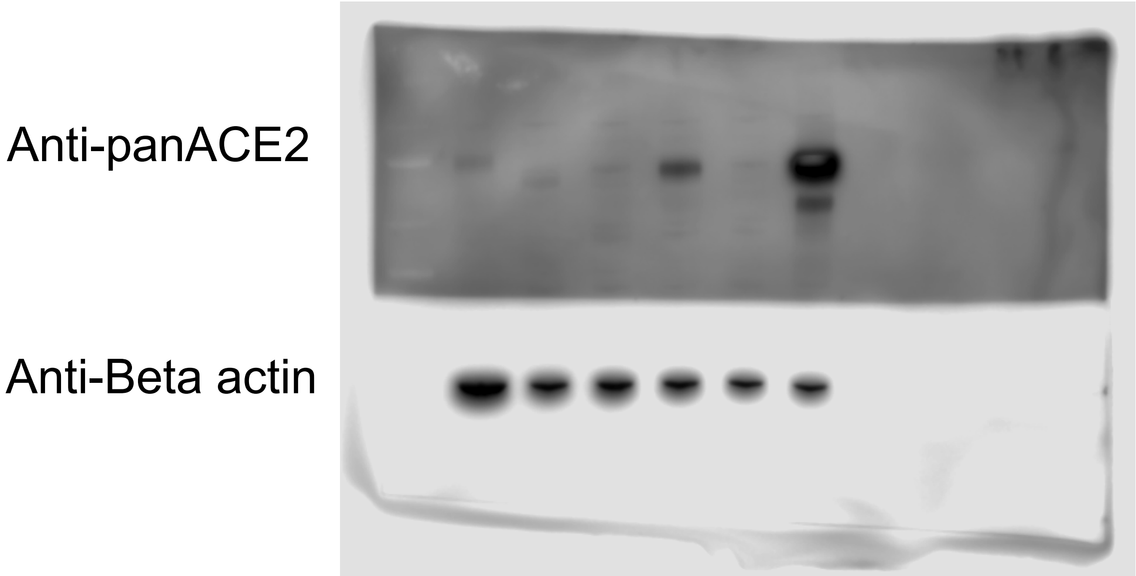

Figure 1F

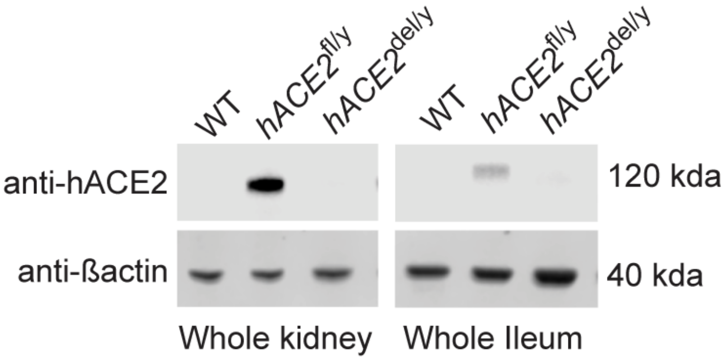
WT
hACE2fl/y
hACE2del/y
WT
hACE2fl/y
hACE2del/y
anti-hACE2
anti-ßactin
120 kda
40 kda
Whole kidney
Whole Ileum

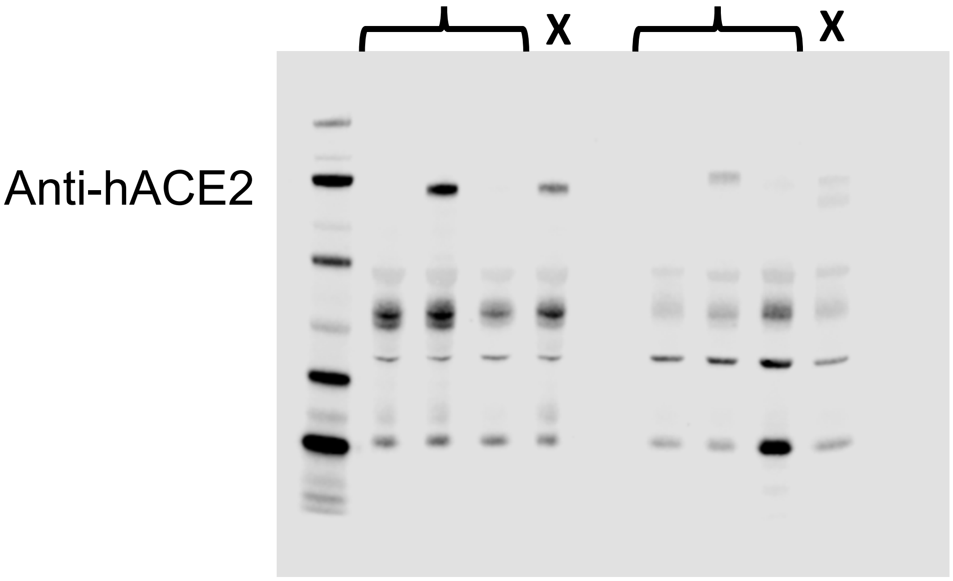
X
X
Anti-hACE2

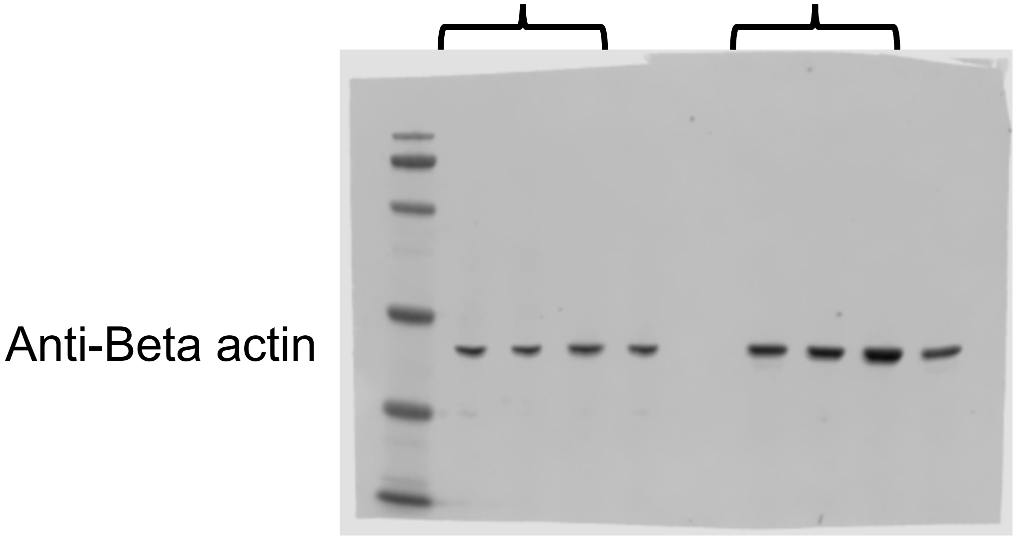
Anti-Beta actin

Figure 1G

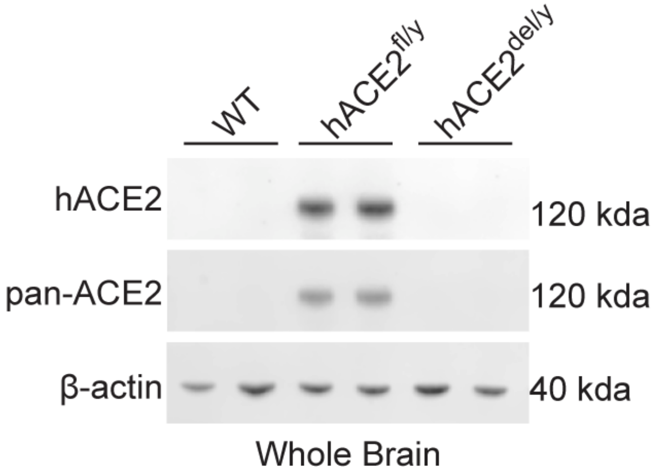

WT
hACE2fl/y
hACE2del/y
hACE2
120 kda
pan-ACE2
120 kda
β-actin
40 kda
Whole Brain

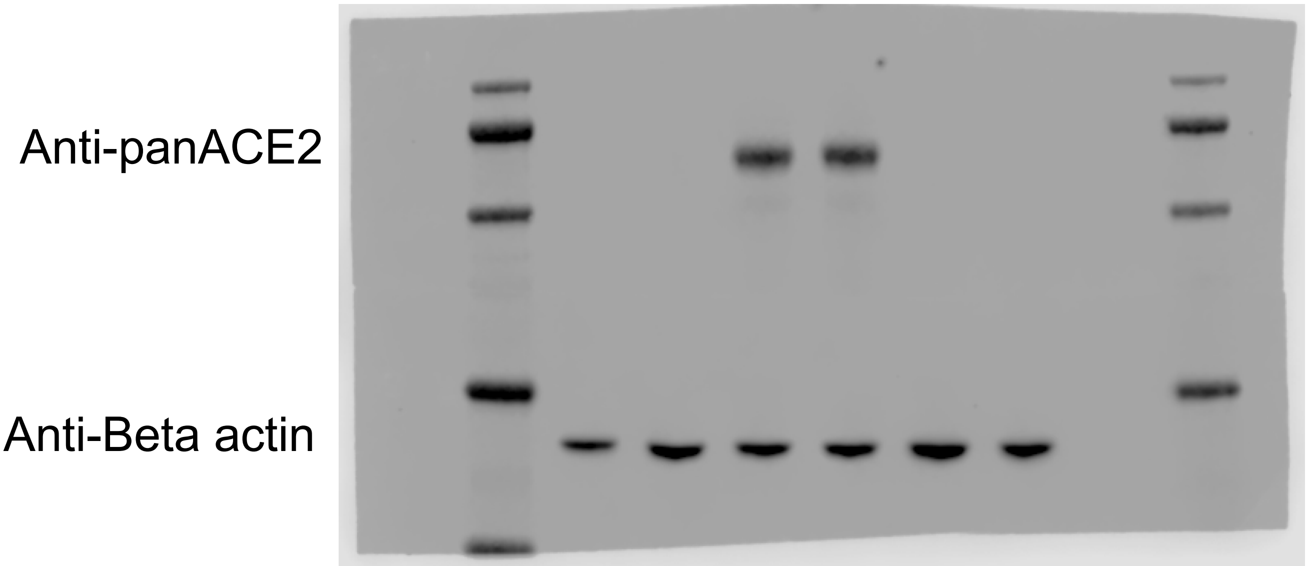

Anti-panACE2
Anti-Beta actin

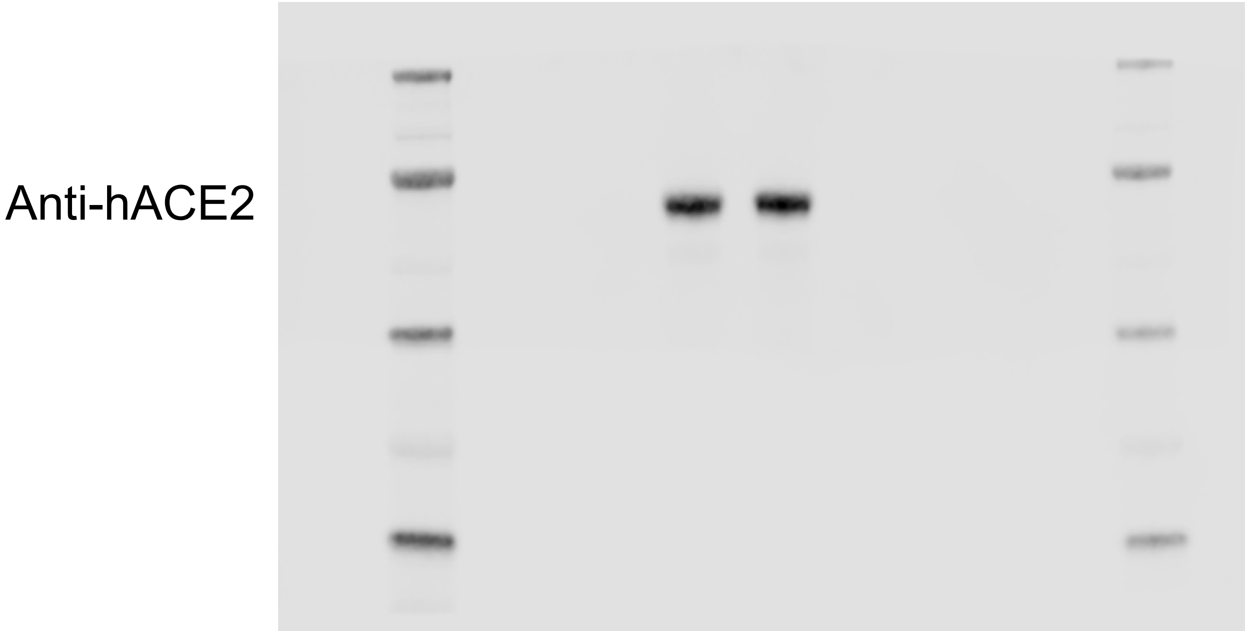

Anti-hACE2

Figure 7D

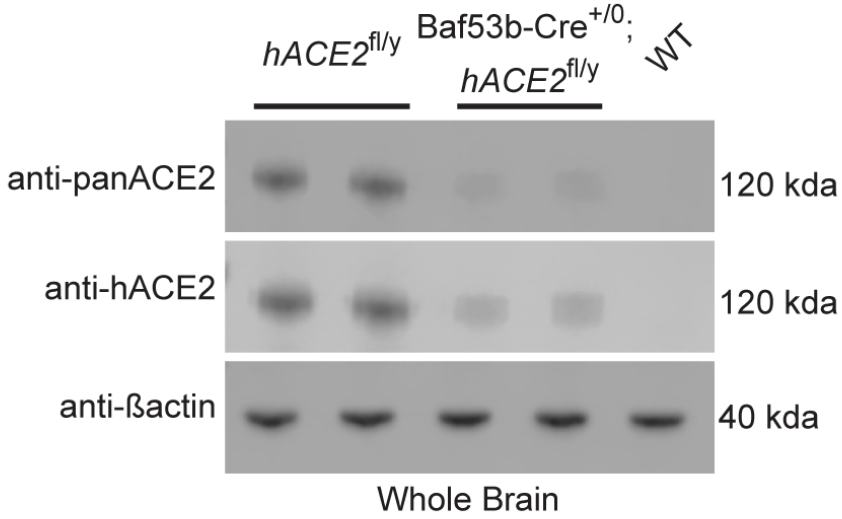

Anti-panACE2

Anti-Beta actin

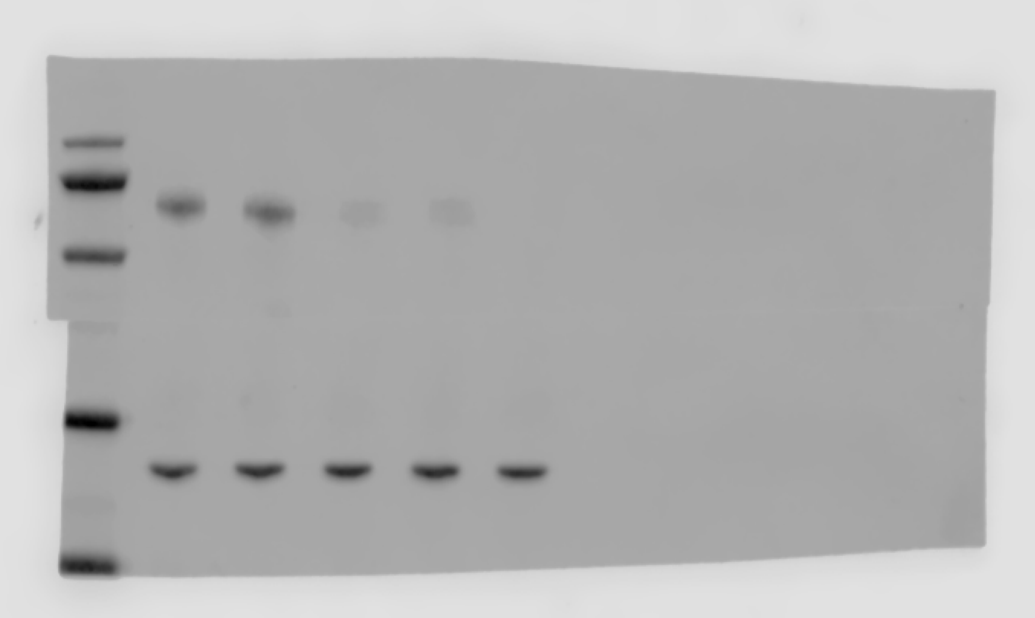

Anti-hACE2

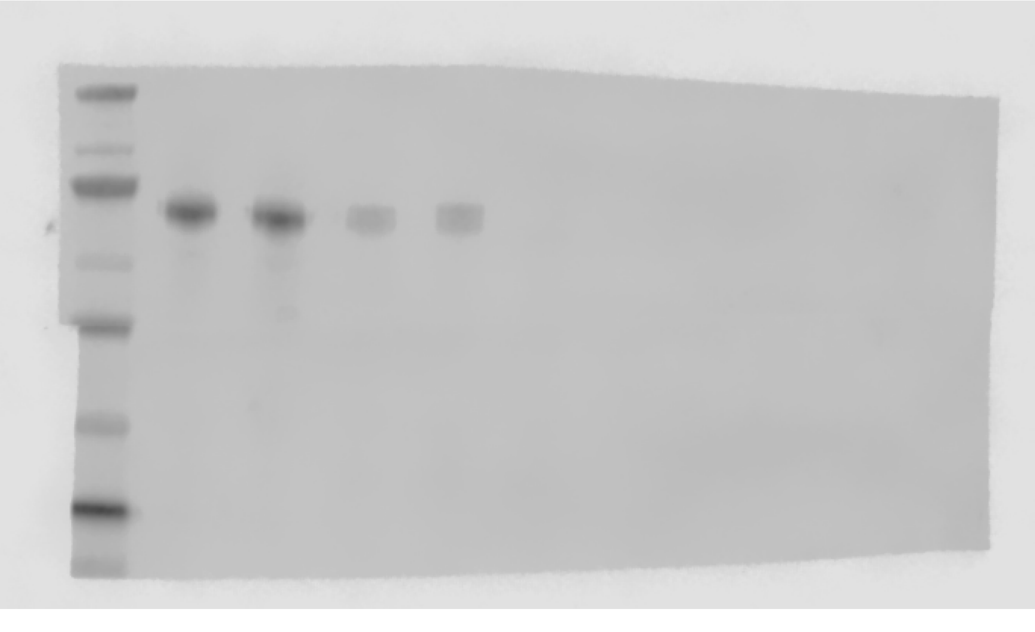

Figure 8B

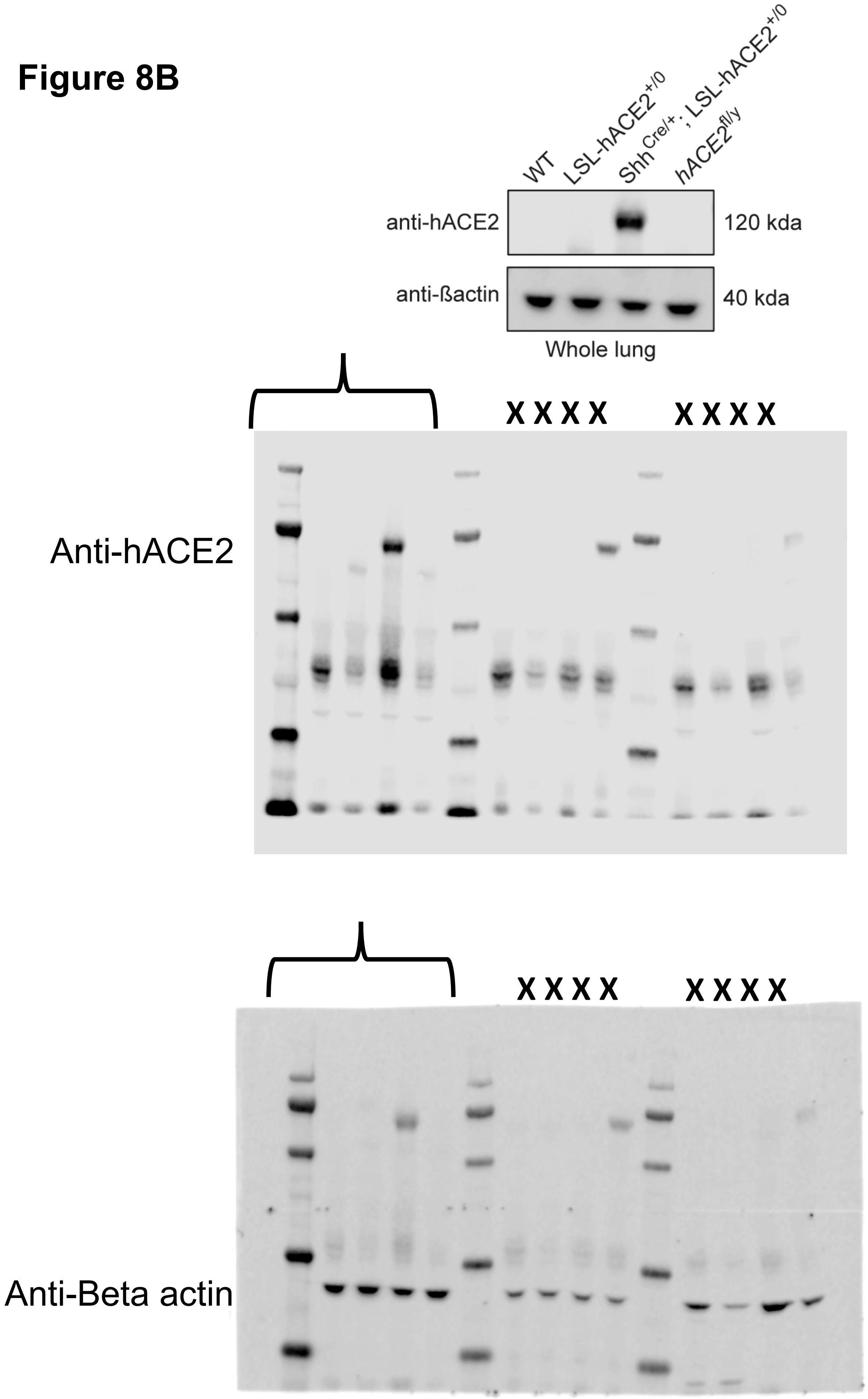
WT
LSL-hACE2+/0
ShhCre/+; LSL-hACE2+/0
hACE2fl/y
anti-hACE2
120 kda
anti-ßactin
40 kda
Whole lung
X X X X
X X X X
Anti-hACE2
X X X X
X X X X
Anti-Beta actin
