# Supplementary material for: Cell-autonomous requirement for ACE2 across organs in lethal mouse SARS-CoV-2 infection
Source: PLoS Biol. 2023 Feb 6;21(2):e3001989. doi: 10.1371/journal.pbio.3001989 (PMC9934376; doi:10.1371/journal.pbio.3001989)
Supplement: Attachment — Submitted filename: Response to the reviewers.docx [file pbio.3001989.s019.docx]

**Response to the reviewers**

Our paper was assessed by Dr. Paula Jauregui and the editorial staff using reviews from another journal. We therefore do not have a response to reviewers in this revision.
